# Multifunctional Polyoxometalate Platforms for Supramolecular Light‐Driven Hydrogen Evolution**

**DOI:** 10.1002/chem.202103817

**Published:** 2021-11-11

**Authors:** Salam Maloul, Matthias van den Borg, Carolin Müller, Linda Zedler, Alexander K. Mengele, Daniel Gaissmaier, Timo Jacob, Sven Rau, Benjamin Dietzek‐Ivanšić, Carsten Streb

**Affiliations:** ^1^ Institute of Inorganic Chemistry I Ulm University Albert-Einstein-Allee 11 89081 Ulm Germany; ^2^ Institute of Electrochemistry Ulm University Albert-Einstein-Allee 47 89081 Ulm Germany; ^3^ Institute of Physical Chemistry Friedrich Schiller University Jena Helmholtzweg 4 07743 Jena Germany; ^4^ Leibniz Institute of Photonic Technologies (IPHT) Albert-Einstein-Straße 9 07745 Jena Germany; ^5^ Helmholtz-Institute Ulm (HIU) Electrochemical Energy Storage Helmholtzstr. 11 89081 Ulm Germany; ^6^ Karlsruhe Institute of Technology (KIT) P.O. Box 3640 76021 Karlsruhe Karlsruhe Germany

**Keywords:** hydrogen evolution, organic-inorganic hybrid, polyoxometalate, self-assembly, supramolecular

## Abstract

Multifunctional supramolecular systems are a central research topic in light‐driven solar energy conversion. Here, we report a polyoxometalate (POM)‐based supramolecular dyad, where two platinum‐complex hydrogen evolution catalysts are covalently anchored to an Anderson polyoxomolybdate anion. Supramolecular electrostatic coupling of the system to an iridium photosensitizer enables visible light‐driven hydrogen evolution. Combined theory and experiment demonstrate the multifunctionality of the POM, which acts as photosensitizer/catalyst‐binding‐site^[1]^ and facilitates light‐induced charge‐transfer and catalytic turnover. Chemical modification of the Pt‐catalyst site leads to increased hydrogen evolution reactivity. Mechanistic studies shed light on the role of the individual components and provide a molecular understanding of the interactions which govern stability and reactivity. The system could serve as a blueprint for multifunctional polyoxometalates in energy conversion and storage.

## Graphical abstract

**Supramolecular visible light‐driven** hydrogen evolution by a covalently linked POM/Pt‐complex is reported. Electrostatic interactions in solution between the anionic POM and a cationic Ir‐based photosensitizer are utilized to enable electron‐transfer and H_2_ evolution. Reactivity enhancement by ligand exchange on the Pt‐complex is possible. Photophysical and theoretical studies shed light on the underlying reaction mechanisms.

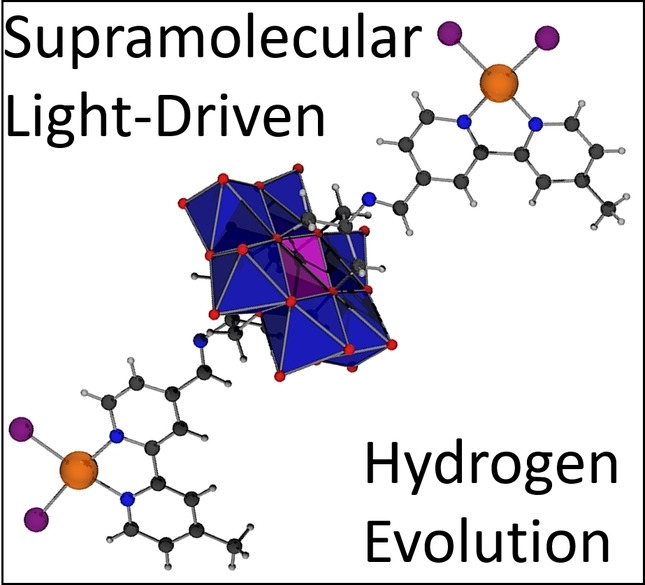

## Introduction

Supramolecular systems combine molecular‐level control of structure and reactivity with the ability to deploy a range of specific intermolecular interactions to access new function. These concepts have led to groundbreaking developments ranging from supramolecular surface patterning^[2]^ to molecular machines^[3]^ and stimuli‐responsive nanostructures.^[4]^ In the field of catalysis, supramolecular concepts have been developed to tune reactivity,^[1]^ selectivity as well as *in situ* catalyst assembly,[^5^, ^6^] highlighting the enormous potential of this field of research.

Over the last decade, pioneering studies have demonstrated the potential of supramolecular catalysis for energy conversion and storage,[^7^, ^8^] for example in the fields of water oxidation,^[9]^ CO_2_ reduction,^[10]^ and the hydrogen evolution reaction (HER).[^11^, ^12^] In light‐driven HER catalysis, pioneering studies have demonstrated how supramolecular systems can be accessed by combining molecular catalysts with molecular photosensitizers. Often, component interactions are controlled by electrostatics, hydrogen bonding or π‐stacking.[^13^, ^14^, ^15^]

The design of molecular platforms is a central concept in supramolecular energy conversion, including HER, as it enables the merging of several functions in one molecule.^[16]^ So‐called photochemical dyads utilize these concepts to combine photosensitizer, charge‐transfer system and HER catalyst in one (supra)molecular assembly.[^17^, ^18^] One classical approach is the linkage of molecular photosensitizer (e. g. Ru‐[^14^, ^19^, ^20^] or Ir‐complexes) and metal complex catalysts (e. g. Pt‐,^[21]^ Pd‐,[^22^, ^23^] Rh‐^[14]^ or Co‐based[^15^, ^20^]) using ditopic bridging ligands for charge‐transfer.^[24]^ This has led to unique models, where insights into the photophysical and electronic properties as well as HER activity have become possible using combined experiment and theory.[^14^, ^19^, ^24^] This concept has been significantly expanded by recent studies where several photosensitizers were linked to HER catalytic sites, leading to operational supramolecular HER systems.[^25^, ^26^, ^27^] Component optimization by chemical modification demonstrated the tunability of these systems, leading to in‐depth understanding of function and reactivity limitations.[^14^, ^21^, ^23^]

In recent years, the concept of supramolecular dyads has been expanded to the field of molecular metal oxides, or polyoxometalates (POMs). POMs are versatile dyad components, which can act as oxidation and reduction catalysts as well as charge separation^[28]^ and charge‐storage sites.[^29^, ^30^] In addition, their structure and reactivity can be tuned over a wide range by chemical modification.[^31^, ^32^, ^33^, ^34^, ^35^] This versatility has made POMs a research focus for advanced, light‐driven energy conversion and storage.[^30^, ^36^, ^37^, ^38^] In particular, the covalent organo‐functionalization of POMs has paved the way for the design of multifunctional platform molecules,[^39^, ^40^] where several functions can in principle be incorporated in one POM.[^32^, ^33^, ^41^, ^42^] This has led to ground‐breaking studies in light‐driven hydrogen evolution,[^27^, ^43^] photo‐electrochemistry^[28]^ and nanocomposites for reversible electron storage.^[44]^ Building on these pioneering studies, we now report the use of POMs as multifunctional redox‐active platform molecules capable of charge storage, HER catalyst anchoring as well as supramolecular photosensitizer binding.^[45]^ Using this approach, multiple functions can be combined in one molecular assembly, while independent reactivity tuning would be possible by modification of each component.

Here, we report a supramolecular approach to light‐driven HER: an anionic Anderson‐type platform‐POM is covalently functionalized with a Pt(II)‐complex HER catalyst. The compound is then electrostatically coupled in solution with a cationic iridium‐photosensitizer (PS) to give the full supramolecular assembly. We demonstrate that in homogeneous solution, the system is capable of light‐driven hydrogen evolution. Experimental and theoretical mechanistic studies show that the POM acts as PS‐ and catalyst‐binding site, facilitating electron transfer under irradiation.

## Results and Discussion

### Catalyst synthesis and characterization

Briefly, the POM‐platform molecules were synthesized in a one‐pot reaction, where the 2,2’‐bipyridine (bpy)‐functionalized Anderson‐anion precursor **POM‐bpy** (*n*Bu_4_N)_3_[MnMo_6_O_18_{(OCH_2_)_3_CNCH(C_11_H_9_N_2_)}_2_]^[43]^ was reacted with the respective Pt‐precursor ([Pt(DMSO)_2_Cl_2_]^[46]^ or [Pt(DMSO)_2_I_2_],^[47]^ in water‐free, de‐aerated acetonitrile to give the POM‐catalyst systems **POM‐PtCl** (*n*Bu_4_N)_3_[MnMo_6_O_18_{(OCH_2_)_3_CNCH(C_11_H_9_N_2_)PtCl_2_}_2_] and **POM‐PtI** (*n*Bu_4_N)_3_[MnMo_6_O_18_{(OCH_2_)_3_CNCH(C_11_H_9_N_2_)PtI_2_}_2_], see Supporting Information for synthetic and analytical details. The solid, orange products were re‐dissolved in N,N‐dimethyl formamide (DMF), and diffusion of EtOAc into the DMF solution gave orange crystals suitable for single‐crystal X‐ray diffraction (scXRD). Yields: 70.2 % (**POM‐PtCl)**, 76.4 % (**POM‐PtI**), yields based on Pt. **POM‐PtCl** and **POM‐PtI** were fully characterized by elemental analysis, NMR‐, FTIR‐spectroscopy and scXRD, see Supporting Information for details (CCDC reference numbers: 2099459 (**POM‐PtCl**) and 2099460 (**POM‐PtI**)). Note that when **POM‐PtCl** and **POM‐PtI** are discussed collectively, we will refer to them as **POM‐PtX**.

### Light‐driven hydrogen evolution

The visible‐light‐driven HER activity of **POM‐PtI** and **POM‐PtCl** was examined using the photosensitizer [Ir(ppy)_2_(bpy)]PF_6_ (**PS**). **PS** was chosen due to its cationic charge, and the literature‐known ability for light‐driven electron transfer to POMs.[^27^, ^48^] In addition, (spectro‐)electrochemical analyses of both **POM‐PtX** species showed, that at least two electron transfers from the photoreduced **PS** (*E*_1/2_ ca. −1.8 V vs. Fc^+^/Fc)^[49]^ to **POM‐PtX** is thermodynamically possible, as both **POM‐PtX** systems feature two redox‐processes at potentials more positive than −1.8 V (see Supporting Information, Table S2, Figures S3, S4).

Thus, light‐driven catalytic studies were performed in water‐free, de‐aerated DMF solutions containing the respective catalyst **POM‐PtX** (12.5 μM), the photosensitizer **PS** (125 μM) and triethyl amine/acetic acid (TEA (1.0 M)/HAc (0.2 M)) as sacrificial proton/electron donors; this experimental setup has been adapted from earlier POM‐based HER studies.[^48^, ^50^, ^51^, ^52^] The samples were irradiated under an Ar atmosphere using monochromatic LEDs (λ_max_=470 nm, *P* ∼40 mW cm^−2^) at 25 °C. Hydrogen evolution was quantified by calibrated gas chromatography. Each data‐point was recorded in triplicate, and averaged turnover numbers (*TONs*) are given based on the molar amount of Pt present, i. e., *TON*=*n*(H_2_)/*n*(Pt). The non‐modified catalysts [Pt(bpy)Cl_2_]/ [Pt(bpy)I_2_] (25 μM) together with **PS** (125 μM) were used as references. Also, control experiments without irradiation or in the absence of POM‐catalysts show virtually no H_2_ evolution (Supporting Information, Figure S5).

**Figure 1:**
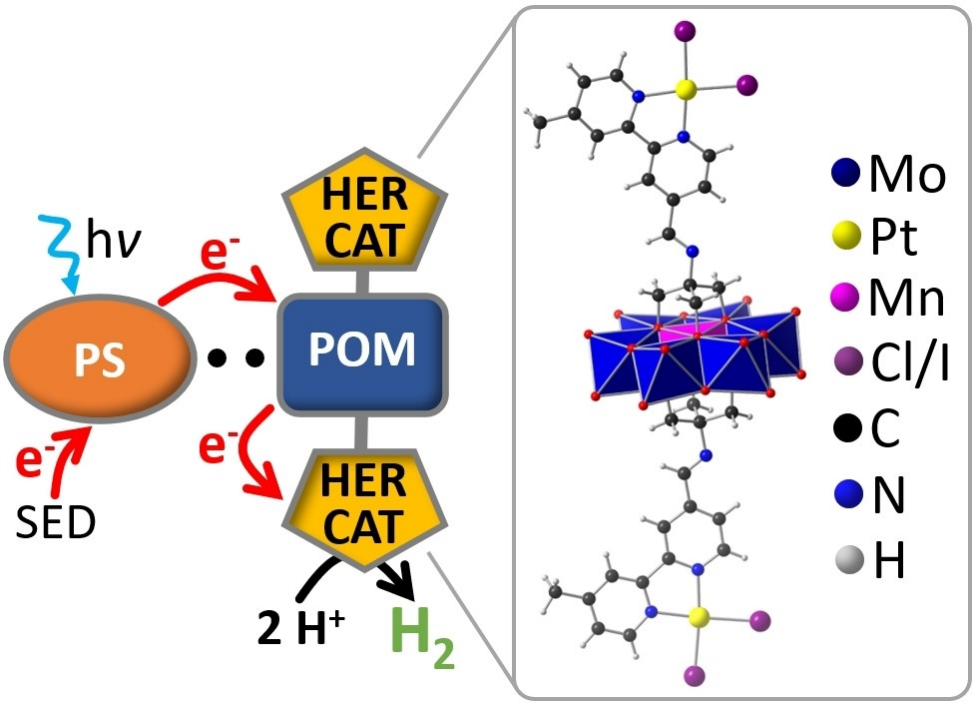
**Left**: Schematic illustration of the supramolecular photochemical system for visible light‐driven hydrogen evolution. An anionic POM‐platform molecule is covalently functionalized with a molecular Pt‐HER catalyst. The system is electrostatically coupled to a cationic photosensitizer (**PS**). SED – sacrificial electron‐donor. **Right**: molecular structure of the **POM‐PtX**‐platform (X=Cl, I, shown here: X=I, for crystallographic data see Supporting Information).

**Figure 2:**
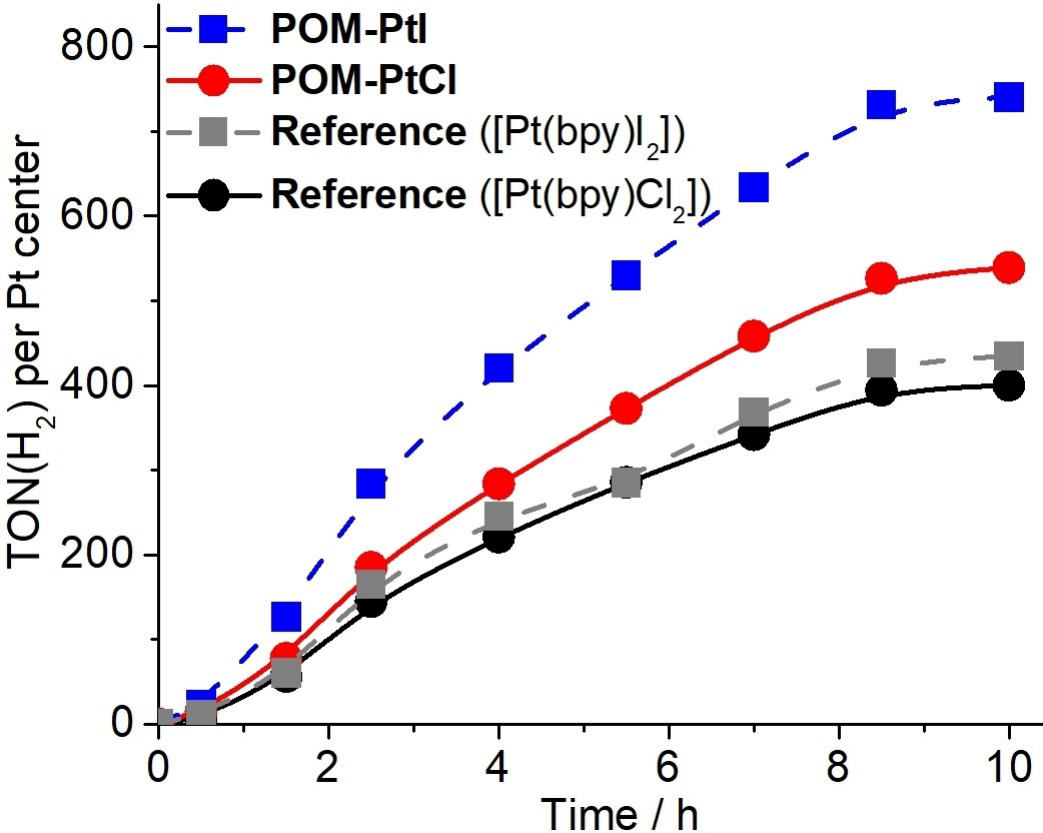
Light‐driven HER activity of **POM‐PtCl** (red), **POM‐PtI** (blue) and the reference compounds [Pt(bpy)Cl_2_] (black) and [Pt(bpy)I_2_] (gray), showing the hydrogen evolution *TON* (calculated per Pt center) over time. Conditions: solvent: water‐free, de‐gassed DMF containing TEA (1.0 M) and HAc (0.2 M). *c*(**POM‐PtX)**=12.5 μM; *c*([Pt(bpy)X_2_])=25 μM, c(**PS**)=125 μM, irradiation: LED, λ_max_=470 nm, P ∼40 mW cm^−2^.

First, the HER activity of **POM‐PtCl** and the reference [Pt(bpy)Cl_2_] were studied. It was observed that under irradiation H_2_ evolution increases linearly with time for both catalysts, see Figure 2. After *t*_irradiation_=10 h, notable reactivity differences are observed. For **POM‐PtCl** the *TONs* reached ∼540, while the reference [Pt(bpy)Cl_2_] only exhibits a *TON* of ca. 400.

To further enhance the activity of the catalyst, the chloride ions on the Pt centers were replaced with iodide to give the **POM‐PtI** cluster (see synthetic discussion above for details). When employed for light‐driven HER under identical conditions as described above, **POM‐PtI** showed significantly higher activity with a maximum *TON* of ca. 740 after *t*_irradiation_=10 h (Figure 2). Note that the higher reactivity of **POM‐PtI** compared with **POM‐PtCl** is in line with previous reports: for the related compounds ([Ru(tbbpy)_2_(tpphz)PtX_2_](PF_6_)_2_ (X=Cl or I), a similar increase of HER activity is found when the chloride ligands are replaced by iodides.^[21]^ The authors assigned this increased activity to the higher electron density at the Pt center due to the large, easily polarized iodide ligands.^[21]^ To the best of our knowledge, this is the first time that ligand‐tuning concepts have been demonstrated in POM‐based HER catalysis.

Based on the above data, we propose that the increased activity of **POM‐PtCl** and **POM‐PtI** compared to the references is due to enhanced electrostatic attraction between the cationic **PS** and the anionic POM‐catalyst. This could enable aggregation and charge‐transfer in solution. To test this hypothesis, we performed a combined experimental and theoretical study which explores the interactions between **PS** and **POM‐PtX**. Due to the higher reactivity, the study used **POM‐PtI** as model.

### Mechanistic photophysical studies

**Figure 3:**
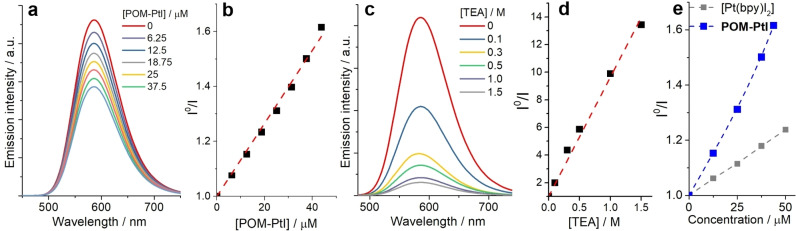
(a) Emission quenching spectra of **PS** ([Ir(ppy)_2_(bpy)]^+^, 125 μM) as a function of [**POM‐PtI**], and (b) corresponding Stern‐Volmer plot; (c) emission quenching spectra of **PS** ([Ir(ppy)_2_(bpy)]^+^, 125 μM) as a function of [TEA], and (d) corresponding Stern‐Volmer plot; (e) Stern‐Volmer plot, comparing the emission quenching of **PS** by **POM‐PtI** and the [Pt(bpy)I_2_] reference. Conditions: water‐free, de‐aerated DMF, details see Supporting Information, Section 7.

To assess the supramolecular interactions between photosensitizer and catalyst, we examined the emission quenching of **PS** by the electron acceptor **POM‐PtI** or the sacrificial electron donor TEA (Figure 3a–d):^[53]^ The steady‐state emission spectra and emission lifetimes of **PS** (λ_excitation_=420 nm) were measured upon addition of TEA or **POM‐PtI** to a DMF solution of **PS** (see Figure 3a,c and Supporting Information, Figure S7). The corresponding Stern‐Volmer plots (Figure 3b,d and Supporting Information, Figures S7, S8) are analyzed by linear fitting resulting in quenching rate constants (k_q_) of 7.1×10^7^ M^−1^s^−1^ (reductive quenching, TEA) and 8.4×10^10^ M^−1^s^−1^ (oxidative quenching, **POM‐PtI**). The calculated mutual diffusion rate constants (k_D_) in DMF are 7.7×10^9^ M^−1^s^−1^ (for **PS**/TEA) and 8.2×10^9^ M^−1^s^−1^ (for **PS**/**POM‐PtI**), respectively (see Supporting Information, Section 7). Comparison between k_D_ and k_q_ however shows that for reductive quenching (PS*/TEA) k_D_ is ∼100 times higher than k_q_, indicating a quenching efficiency per encounter of ca. 1 %.^[54]^ In contrast, for the oxidative quenching (PS*/**POM‐PtI**) k_D_ is ∼10 times smaller than k_q_, indicating a quenching efficiency per encounter of formally >100 %. Two interpretations are in line with these observations: (a) the interactions of the **PS** cation and the **POM‐PtI** anion increase the rate of interaction beyond the frequency calculated for neutral reaction partners within the Collins‐Kimball model (see Supporting Information, Section 7);^[55]^ (b) a supramolecular pre‐assembly of cationic **PS** and anionic **POM‐PtI** enables PS* quenching at significantly faster rates than the mutual diffusion, i. e., in the limit of static quenching.^[56]^ As a result, the oxidative quenching of PS* has to occur via both, static and dynamic quenching.

Next, the quenching efficiency of **PS** by the anionic **POM‐PtI** was compared with the quenching behavior of the charge‐neutral [Pt(bpy)I_2_] reference (Figure 3e). Here, **POM‐PtI** showed a significantly higher quenching efficiency compared to the reference compound. This supports the suggestion that electrostatic attraction of **PS** and **POM‐PtI** in solution can be used to enhance electronic interactions of the reaction partners following photoexcitation of the **PS**. This observation also supports the HER catalytic findings, where **POM‐PtI** shows significantly higher reactivity than the [Pt(bpy)I_2_] reference (Figure 2).

### Theoretical studies of PS/POM‐PtI aggregation

To better understand the coupling between **PS** and **POM‐PtCl** and its effects on the electronic properties of the system, first principles simulations using spin‐polarized density functional theory (DFT) calculations within the ORCA program package were performed.^[57]^ Initial geometry optimization used the PBEh‐3c composite method^[58]^ followed by geometry optimizations with the PBE0 hybrid functional[^59^, ^60^] and a def2‐TZVP basis set.^[61]^ For molybdenum and iridium, effective core potentials were used to replace the core electrons.[^62^, ^63^] Dispersion interactions were accounted for by atom‐pairwise correction with the Becke–Johnson damping scheme [D3(BJ)].[^64^, ^65^] The conductor‐like polarizable continuum model (CPCM) was used to implicitly include the DMF solvent (ϵ =38.30 and n =1.43).^[66]^ Electron densities were analyzed by Hirshfeld population analysis to determine atomic charges.^[67]^

**Figure 4:**
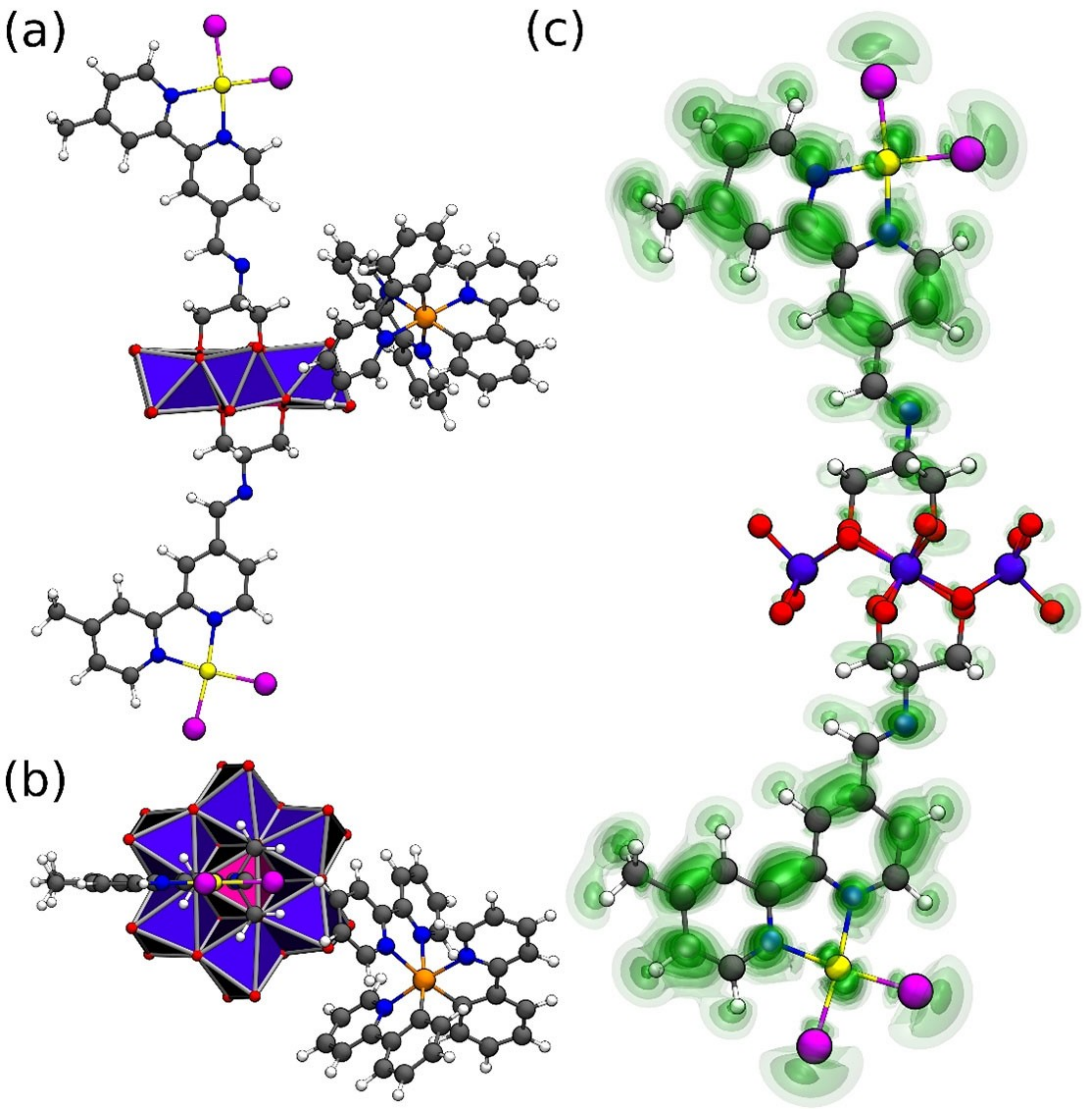
Energetically most favored interaction sites between **PS** and **POM‐PtI** in (a) side view and (b) top view; (c) representation of the $f^{+}\;$ Fukui function calculated for the one‐electron reduced **POM‐PtCl**, shown as charge‐difference plot. The iso‐surfaces correspond to electron‐accepting regions where an additional added electron will be located (as indicated by the $f^{+}$ Fukui function). Regions with higher values are marked in darker shades of green. Molecular color scheme, see Figure 1.

To gain insights into the energetics and binding behavior of **PS** and **POM‐PtX**, we used DFT calculations to assess the interaction energies between both species. As model systems, we focused on **PS** (charge: 1+) and **POM‐PtCl** (charge: 3‐), see Figure 4. As references, we studied [Pt(bpy)Cl_2_] (charge‐neutral) and the TRIS‐functionalized prototype Anderson anion [MnMo_6_O_18_{(CH_2_O)_3_CNH_2_}_2_]^3−^ (**POM‐Ref**, charge: 3‐, Supporting Information, Figure S9).^[43]^ Initial studies gave interaction energies of −48 kJ/mol (**PS**/[Pt(bpy)Cl_2_]) and −76 kJ/mol (**PS**/**POM‐Ref**) and −77 kJ/mol (**PS**/**POM‐PtCl**), respectively. This highlights, that supramolecular aggregation in solution between photosensitizer and POM is thermodynamically favored. For the **PS**/**POM‐PtCl** system, we observe that in the energetically most favored system, **PS** is located next to the metal oxo cluster (Figure 4), highlighting the importance of the POM for **PS**‐binding. Note that quantitatively virtually identical results were obtained for **POM‐PtI**; these are shown in the Supporting Information, Section 8).

Next, we theoretically assessed the electronic consequences of an electron‐transfer from **PS** to **POM‐PtCl**, which is a key redox step of the HER catalytic cycle. To this end, single‐point calculations were performed on the one‐electron reduced **POM‐PtCl** as well as the one‐electron reduced reference systems (i. e., [Pt(bpy)Cl_2_] and **POM‐Ref**). This allowed us to compare the Hirshfeld net charges of the one‐electron reduced systems with the charge‐distribution of the non‐reduced systems. Note that this procedure is analogous to determining condensed Fukui functions.[^68^, ^69^] While Fukui functions are often used as descriptors for the chemical reactivity of a molecule (with respect to its electrophilic and nucleophilic regions), here, the function for a nucleophilic attack will serve as a measure for the uptake and distribution of additional electron density in the systems studied. The Fukui function $f^{+}$ is given by the difference between the electron densities of the initial state $\rho \left(N\right)$ and the state featuring one additional electron ρ(N+1) :

$$f^{+}=\;\rho \left(N+1\right)+\;\rho \left(N\right)$$

The corresponding condensed Fukui function $f_{k}^{+}$ is defined as:

$$f_{k}^{+}=q_{k}\left(N+1\right)+q_{k}\left(N\right)$$

where $q_{k}\left(N\right)$ describes the atomic charges at the respective atomic center k of the original system and $q_{k}\left(N+1\right)$ those of the system with one additional electron. After normalization, this provides a percentage value of the additional electron density at any given atom. For clarity, we have combined the resulting atomic percentages into three molecular sub‐units, i. e. the 2,2’‐bipyridine ligands (bpy), the platinum‐chloride fragment {PtCl_2_} and the POM metal‐oxo framework {MnMo_6_O_24_}, see Table 1 and Supporting Information, Section 8 for computational details.

**Table 1:** Percentage distribution of an additional electron taken up by **POM‐PtCl**, **POM‐Ref** and [Pt(bpy)Cl_2_] as calculated using normalized nucleophilic Fukui functions $f_{k}^{+}$ and Hirshfeld population analysis.

**Fragment**	**POM‐PtCl**	**POM‐Ref**	**[Pt(bpy)Cl_2_]**
bpy	75.5	–	79.7
{PtCl_2_}	18.8	–	20.3
{MnMo_6_O_24_}	5.7	88.6	–

As shown in Table 1, analysis of the nucleophilic Fukui function for the one‐electron reduced **POM‐PtCl** indicates that additional electron density will be distributed on the Pt‐HER catalyst, either on the {PtCl_2_} fragment (18.8 %) or the bpy ligand (75.5 %), while only a low percentage of the additional electron is distributed on the POM framework (5.7 %). For the reference systems, the expected distributions are observed: in **POM‐Ref**, 88.6 % of the electron density is located on the POM framework, while in [Pt(bpy)Cl_2_], 79.7 % are located on the bpy and 20.3 % on the {PtCl_2_} fragment (Figure 4 and Supporting Information, Table S3). This is in line with experimental electrochemical and spectro‐electrochemical data, which indicate that an electron transfer to the one‐electron reduced **POM‐PtX**, i. e., a second reduction of **POM‐PtX** occurs on the {Pt(bpy)X_2_} moiety (Supporting Information, Figures S3, S4).

**Figure 5:**
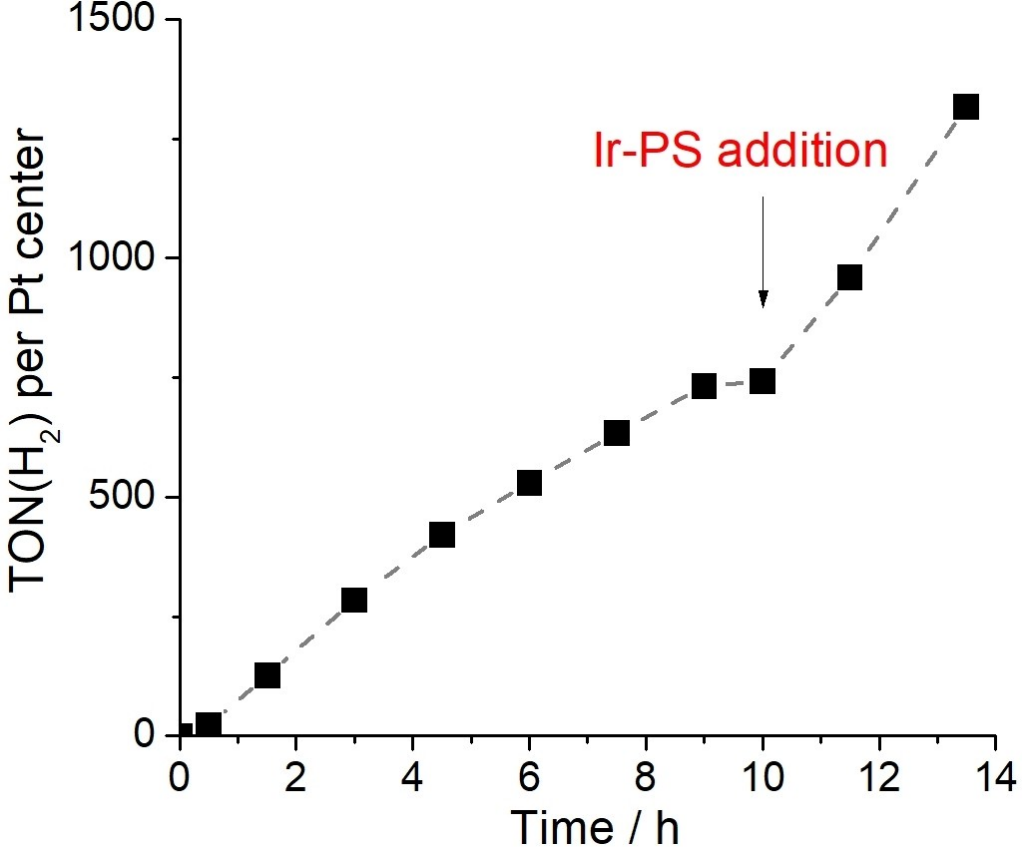
Reusability of the catalyst **POM‐PtI** demonstrated by adding an aliquot of **PS** after HER activity ceased (after *t*_irradiation_=10 h). Conditions are identical to the HER experiments shown in Figure 2

**Reusability and stability of the molecular catalyst**: Finally, we were interested in understanding the causes of the loss of HER activity observed after ∼9 h of irradiation (Figure 2). We hypothesized (based on initial UV‐Vis spectroscopy of the reaction solution, Supporting Information, Figure S10), that this could be due to **PS** degradation, and thus, loss of the light‐harvesting capabilities of the system. In contrast, prolonged visible light‐irradiation of **POM‐PtX** resulted in minor UV‐Vis absorption changes in the 400–450 nm region. This could indicate a very slow degradation (Supporting Information, Figure S11), which is currently being studied by *in situ* optical and vibrational spectroscopies. To experimentally demonstrate that **PS** degradation is the main deactivation pathway under the given conditions, we performed a reusability experiment where the standard photochemical experiment (identical to the experiment shown in Figure 2, catalyst: **POM‐PtI**) was performed. After 10 h of irradiation, an aliquot of fresh **PS** (110 μM) was added to the reaction mixture and irradiation was resumed. As illustrated in Figure 5, the system resumed hydrogen evolution at high reactivity. This observation provides evidence that **PS** degradation is a major factor in reactivity loss in the system described, while catalytic activity by the **POM‐PtI** is still observed. Thus, future studies can explore the design of more robust photosensitizers with optimized POM binding behavior.^[70]^

To gain further insight into the stability of **POM‐PtX**, we performed a range of mechanistic analyses: First, UV‐Vis spectroscopy of **POM‐PtX** in the reaction solvent DMF showed no spectral changes over 18 h (Supporting Information, Figure S11). Also, microfiltration and UV‐Vis spectroscopic analysis of a reaction mixture containing **POM‐PtI** and **PS** (molar ratio: 1/10) showed no spectral changes, indicating that no colloidal particles are observed (Supporting Information, Figure S12). This was supported by dynamic light scattering (DLS) on the catalytic reaction solution, where no particles within the measurement range (1–10000 nm) were detected. This suggests that the system does not produce colloidal platinum particles as the active HER catalyst, but that a molecular system operates as HER catalyst in the present study. Note that the “classical” mercury amalgam test for Pt colloids was not suitable for the present system, as elemental Hg undergoes redox‐reactions with **POM‐PtX** already in the dark, resulting in fast degradation of the **POM‐PtX** molecule. Similar behavior has been reported before.^[21]^

To provide further evidence for the molecular nature of the HER system reported, we performed a catalytic experiment under the standard conditions described in Figure 2 using **POM‐PtCl** as model catalyst. Then, excess *n*Bu_4_NI was added to the reaction solution to trigger iodide‐for chloride ligand exchange on **POM‐PtCl** (leading to partial *in situ* formation of **POM‐PtI**). As predicted from our initial experiments (Figure 2), this led to an increase of the *TON* (+∼20 %), thereby providing further evidence of a molecular HER catalysis (Supporting Information, Figure S13).[^71^, ^72^]

## Conclusion

In sum, we demonstrate how organo‐functionalized polyoxometalates can be used as multifunctional platforms for supramolecular light‐driven hydrogen evolution catalysis. Electrostatic solution‐interactions between an anionic, Pt‐functionalized POM dyad with a cationic photosensitizer lead to light‐driven charge‐transfer and H_2_ evolution in solution. The reactivity of the dyads surpasses the purely intermolecular reference systems, and reactivity tuning of the dyads is possible by ligand exchange. Experiment and theory shed light on the underlying interactions in solution, and initial stability analyses highlight future areas of development, such as the shift from organic to aqueous solvents. Our results open new avenues for the design of supramolecular light‐driven hydrogen evolution catalysts.

## Experimental Section

Deposition Numbers 2099459 (for **POM‐PtCl**) and 2099460 (for **POM‐PtI**) contain the supplementary crystallographic data for this paper. These data are provided free of charge by the joint Cambridge Crystallographic Data Centre and Fachinformationszentrum Karlsruhe Access Structures service.

## Conflict of interest

The authors declare no conflict of interest.

1

## Supporting information

As a service to our authors and readers, this journal provides supporting information supplied by the authors. Such materials are peer reviewed and may be re‐organized for online delivery, but are not copy‐edited or typeset. Technical support issues arising from supporting information (other than missing files) should be addressed to the authors.

Supporting Information

## Data Availability

The data that support the findings of this study are available from the corresponding author upon reasonable request.

## References

[1] S. Karlsson , J. Boixel , Y. Pellegrin , E. Blart , H.-C. Becker , F. Odobel , L. Hammarström , Faraday Discuss. 2012, 155, 233–252. 22470977 10.1039/c1fd00089f

[2] D. P. Goronzy , M. Ebrahimi , F. Rosei , Arramel , Y. Fang , S. De Feyter , S. L. Tait , C. Wang , P. H. Beton , A. T. S. Wee , et al., ACS Nano 2018, 12, 7445–7481. 30010321 10.1021/acsnano.8b03513

[3] R. Iino, K. Kinbara, Z. (guest eds) Bryant, *Chem. Rev*. **2020**, *120*, 1–460. 10.1021/acs.chemrev.9b00819 31910626

[4] Z. Li , N. Song , Y.-W. Yang , Matter 2019, 1, 345–368.

[5] J. Meeuwissen , J. N. H. Reek , Nat. Chem. 2010, 2, 615–621. 20651721 10.1038/nchem.744

[6] M. Raynal , P. Ballester , A. Vidal-Ferran , P. W. N. M. van Leeuwen , Chem. Soc. Rev. 2014, 43, 1660–1733. 24356298 10.1039/c3cs60027k

[7] B. Zhang , L. Sun , Chem. Soc. Rev. 2019, 48, 2216–2264. 30895997 10.1039/c8cs00897c

[8] T. Keijer , T. Bouwens , J. Hessels , J. N. H. Reek , Chem. Sci. 2021, 12, 50–70. 10.1039/d0sc03715j PMC8179670 34168739

[9] V. Kunz , D. Schmidt , M. I. S. Röhr , R. Mitrić , F. Würthner , Adv. Energy Mater. 2017, 7, 1602939.

[10] Y. Tamaki , O. Ishitani , ACS Catal. 2017, 7, 3394–3409.

[11] F. Gärtner , B. Sundararaju , A. E. Surkus , A. Boddien , B. Loges , H. Junge , P. H. Dixneuf , M. Beller , Angew. Chem. Int. Ed. 2009, 48, 9962–9965; 10.1002/anie.200905115 19937629

[12] A. Neubauer , G. Grell , A. Friedrich , S. I. Bokarev , P. Schwarzbach , F. Gärtner , A.-E. Surkus , H. Junge , M. Beller , O. Kühn , et al., J. Phys. Chem. Lett. 2014, 5, 1355–1360. 26269979 10.1021/jz5004318

[13] D. R. Whang , S. Y. Park , ChemSusChem 2015, 8, 3204–3207. 26315804 10.1002/cssc.201500787

[14] T. A. White , B. N. Whitaker , K. J. Brewer , J. Am. Chem. Soc. 2011, 133, 15332–15334. 21875106 10.1021/ja206782k

[15] J. Dong , M. Wang , P. Zhang , S. Yang , J. Liu , X. Li , L. Sun , J. Phys. Chem. C 2011, 115, 15089–15096.

[16] J. J. Walsh , A. M. Bond , R. J. Forster , T. E. Keyes , Coord. Chem. Rev. 2016, 306, 217–234.

[17] V. Balzani , A. Credi , M. Venturi , ChemSusChem 2008, 1, 26–58. 18605661 10.1002/cssc.200700087

[18] F. Odobel , M. Séverac , Y. Pellegrin , E. Blart , C. Fosse , C. Cannizzo , C. R. Mayer , K. J. Elliott , A. Harriman , Chem. Eur. J. 2009, 15, 3130–3138. 19197929 10.1002/chem.200801880

[19] S. Tschierlei , M. Karnahl , M. Presselt , B. Dietzek , J. Guthmuller , L. González , M. Schmitt , S. Rau , J. Popp , Angew. Chem. Int. Ed. 2010, 49, 3981–3984; 10.1002/anie.200906595 20411552

[20] A. Fihri , V. Artero , M. Razavet , C. Baffert , W. Leibl , M. Fontecave , Angew. Chem. Int. Ed. 2008, 47, 564–567; 10.1002/anie.200702953 18095368

[21] M. G. Pfeffer , T. Kowacs , M. Wächtler , J. Guthmuller , B. Dietzek , J. G. Vos , S. Rau , Angew. Chem. Int. Ed. 2015, 54, 6627–6631; 10.1002/anie.201409442 25858688

[22] S. Rau , B. Schäfer , D. Gleich , E. Anders , M. Rudolph , M. Friedrich , H. Görls , W. Henry , J. G. Vos , Angew. Chem. Int. Ed. 2006, 45, 6215–6218; 10.1002/anie.200600543 16917792

[23] M. G. Pfeffer , B. Schäfer , G. Smolentsev , J. Uhlig , E. Nazarenko , J. Guthmuller , C. Kuhnt , M. Wächtler , B. Dietzek , V. Sundström , S. Rau , Angew. Chem. Int. Ed. 2015, 54, 5044–5048; 10.1002/anie.201409438 25613551

[24] M. Schulz , M. Karnahl , M. Schwalbe , J. G. Vos , Coord. Chem. Rev. 2012, 256, 1682–1705.

[25] M. Elvington , J. Brown , S. M. Arachchige , K. J. Brewer , J. Am. Chem. Soc. 2007, 129, 10644–10645. 17696350 10.1021/ja073123t

[26] S. Chen , K. Li , F. Zhao , L. Zhang , M. Pan , Y.-Z. Fan , J. Guo , J. Shi , C.-Y. Su , Nat. Commun. 2016, 7, 13169. 27827376 10.1038/ncomms13169 PMC5105156

[27] B. Matt , J. Fize , J. Moussa , H. Amouri , A. Pereira , V. Artero , G. Izzet , A. Proust , Energy Environ. Sci. 2013, 6, 1504–1508. 24443654 10.1039/C3EE40352A PMC3890516

[28] F. A. Black , A. Jacquart , G. Toupalas , S. Alves , A. Proust , I. P. Clark , E. A. Gibson , G. Izzet , Chem. Sci. 2018, 9, 5578–5584. 30061989 10.1039/c8sc00862k PMC6048759

[29] B. Rausch , M. D. Symes , G. Chisholm , L. Cronin , Sci. J. 2014, 345, 1326–1330. 10.1126/science.1257443 25214625

[30] L. G. Bloor , R. Solarska , K. Bienkowski , P. J. Kulesza , J. Augustynski , M. D. Symes , L. Cronin , J. Am. Chem. Soc. 2016, 138, 6707–6710. 27159121 10.1021/jacs.6b03187 PMC5033397

[31] Special POM-themed issue, L. Cronin, A. Müller (guest eds.), *Chem. Soc,. Rev*. **2012**, *41*, 7325–7648.

[32] A. V. Anyushin , A. Kondinski , T. N. Parac-Vogt , Chem. Soc. Rev. 2020, 49, 382–432. 31793568 10.1039/c8cs00854j

[33] A. Proust , B. Matt , R. Villanneau , G. Guillemot , P. Gouzerh , G. Izzet , Chem. Soc. Rev. 2012, 41, 7605–7622. 22782306 10.1039/c2cs35119f

[34] K. J. Elliott , A. Harriman , L. Le Pleux , Y. Pellegrin , E. Blart , C. R. Mayer , F. Odobel , Phys. Chem. Chem. Phys. 2009, 11, 8767–8773. 20449021 10.1039/b905548g

[35] A. Harriman , K. J. Elliott , M. a. H. Alamiry , L. Le Pleux , M. Séverac , Y. Pellegrin , E. Blart , C. Fosse , C. Cannizzo , C. R. Mayer , et al., J. Phys. Chem. C 2009, 113, 5834–5842.

[36] C. Streb , Dalton Trans. 2012, 41, 1651. 22183140 10.1039/c1dt11220a

[37] H. Lv , Y. V. Geletii , C. Zhao , J. W. Vickers , G. Zhu , Z. Luo , J. Song , T. Lian , D. G. Musaev , C. L. Hill , Chem. Soc. Rev. 2012, 41, 7572–7589. 22972187 10.1039/c2cs35292c

[38] A. Sartorel , M. Carraro , F. M. Toma , M. Prato , M. Bonchio , Energy Environ. Sci. 2012, 5, 5592–5603.

[39] Y.-F. Song , R. Tsunashima , Chem. Soc. Rev. 2012, 41, 7384. 22850732 10.1039/c2cs35143a

[40] G. Izzet , F. Volatron , A. Proust , Chem. Rec. 2017, 17, 250–266. 27546462 10.1002/tcr.201600092

[41] A. J. Kibler , G. N. Newton , Polyhedron 2018, 154, 1–20.

[42] A. Blazevic , A. Rompel , Coord. Chem. Rev. 2016, 307, Part, 42–64.

[43] S. Schönweiz , S. A. Rommel , J. Kübel , M. Micheel , B. Dietzek , S. Rau , C. Streb , Chem. Eur. J. 2016, 22, 12002–12005. 27418410 10.1002/chem.201602850

[44] E. Hampson , J. M. Cameron , S. Amin , J. Kyo , J. A. Watts , H. Oshio , G. N. Newton , Angew. Chem. Int. Ed. 2019, 58, 18281–18285; 10.1002/anie.201912046 PMC6916258 31595597

[45] M. Bonchio , Z. Syrgiannis , M. Burian , N. Marino , E. Pizzolato , K. Dirian , F. Rigodanza , G. A. Volpato , G. La Ganga , N. Demitri , et al., Nat. Chem. 2019, 11, 146–153. 30510216 10.1038/s41557-018-0172-y

[46] T. A. K. Al-Allaf , L. J. Rashan , A. S. Abu-Surrah , R. Fawzi , M. Steimann , Transition Met. Chem. 1998, 23, 403–406.

[47] T. Kowacs , L. O'Reilly , Q. Pan , A. Huijser , P. Lang , S. Rau , W. R. Browne , M. T. Pryce , J. G. Vos , Inorg. Chem. 2016, 55, 2685–2690. 26925834 10.1021/acs.inorgchem.5b01752

[48] Y. Luo , S. Maloul , M. Wächtler , A. Winter , U. S. Schubert , C. Streb , B. Dietzek , Chem. Commun. 2020, 56, 10485–10488. 10.1039/d0cc04509h 32766633

[49] Y. Ohsawa , S. Sprouse , K. A. King , M. K. DeArmond , K. W. Hanck , R. J. Watts , J. Phys. Chem. 1987, 91, 1047–1054.

[50] S. Schönweiz , M. Heiland , M. Anjass , T. Jacob , S. Rau , C. Streb , Chem. Eur. J. 2017, 23, 15370–15376. 28763122 10.1002/chem.201702116

[51] S. Schönweiz, S. Knoll, M. Anjass, M. Braumüller, S. Rau, C. Streb, **n.d**. 10.1039/c6dt03370a 27711825

[52] Y. Luo , S. Maloul , S. Schönweiz , M. Wächtler , C. Streb , B. Dietzek , Chem. A Eur. J. 2020, 26, 8045–8052. 10.1002/chem.202000982 PMC7383969 32237163

[53] B. M. Hockin , C. Li , N. Robertson , E. Zysman-Colman , Catal. Sci. Technol. 2019, 9, 889–915.

[54] A. Neubauer , G. Grell , A. Friedrich , S. I. Bokarev , P. Schwarzbach , F. Gärtner , A.-E. Surkus , H. Junge , M. Beller , O. Kühn , et al., J. Phys. Chem. Lett. 2014, 5, 1355–1360. 26269979 10.1021/jz5004318

[55] F. C. Collins , G. E. Kimball , J. Colloid Sci. 1949, 4, 425–437.

[56] L. M. Kiefer , K. J. Kubarych , Chem. Sci. 2018, 9, 1527–1533. 29675196 10.1039/c7sc04533f PMC5887230

[57] F. Neese , Wiley Interdiscip. Rev.: Comput. Mol. Sci. 2018, 8, e1327.

[58] S. Grimme , J. G. Brandenburg , C. Bannwarth , A. Hansen , J. Chem. Phys. 2015, 143, 054107. 26254642 10.1063/1.4927476

[59] J. P. Perdew , M. Ernzerhof , K. Burke , J. Chem. Phys. 1996, 105, 9982–9985.

[60] C. Adamo , V. Barone , J. Chem. Phys. 1999, 110, 6158–6170.

[61] F. Weigend , R. Ahlrichs , Phys. Chem. Chem. Phys. 2005, 7, 3297. 16240044 10.1039/b508541a

[62] K. Eichkorn , F. Weigend , O. Treutler , R. Ahlrichs , Theor. Chem. Accounts Theory, Comput. Model. (Theoretica Chim. Acta) 1997, 97, 119–124.

[63] D. Andrae , U. Häußermann , M. Dolg , H. Stoll , H. Preuß , Theor. Chim. Acta 1991, 78, 247–266.

[64] S. Grimme , J. Antony , S. Ehrlich , H. Krieg , J. Chem. Phys. 2010, 132, 154104. 20423165 10.1063/1.3382344

[65] S. Grimme , S. Ehrlich , L. Goerigk , J. Comput. Chem. 2011, 32, 1456–1465. 21370243 10.1002/jcc.21759

[66] M. Cossi , N. Rega , G. Scalmani , V. Barone , J. Comput. Chem. 2003, 24, 669–681. 12666158 10.1002/jcc.10189

[67] F. L. Hirshfeld , Theor. Chim. Acta 1977, 44, 129–138.

[68] K. Fukui , T. Yonezawa , H. Shingu , J. Chem. Phys. 1952, 20, 722–725.

[69] R. G. Parr , W. Yang , J. Am. Chem. Soc. 1984, 106, 4049–4050.

[70] K. Heussner , K. Peuntinger , N. Rockstroh , L. C. Nye , I. Ivanovic-Burmazovic , S. Rau , C. Streb , Chem. Commun. (Camb.) 2011, 47, 6852–6854. 21556406 10.1039/c1cc11859e

[71] S. A. De Pascali , D. Migoni , P. Papadia , A. Muscella , S. Marsigliante , A. Ciccarese , F. P. Fanizzi , Dalton Trans. 2006, 2, 5077–5087. 10.1039/b610945d 17060994

[72] M. González , R. Bartolomé , S. Matarraz , E. Rodríguez-Fernández , J. L. Manzano , M. Pérez-Andrés , A. Orfao , M. Fuentes , J. J. Criado , J. Inorg. Biochem. 2012, 106, 43–45. 22112838 10.1016/j.jinorgbio.2011.08.015

