# Supplementary material for: Multifunctional Polyoxometalate Platforms for Supramolecular Light‐Driven Hydrogen Evolution
Source: Chemistry. 2021 Nov 11;27(68):16846–52. doi: 10.1002/chem.202103817 (PMC9299148; doi:10.1002/chem.202103817)
Supplement: Supplementary file 1 — Supporting Information [file CHEM-27-16846-s001.pdf]

Chemistry–A European Journal

Supporting Information

Multifunctional Polyoxometalate Platforms for Supramolecular Light-Driven Hydrogen Evolution

Chemistry–A European Journal

Supporting Information

Multifunctional Polyoxometalate Platforms for Supramolecular Light-Driven Hydrogen Evolution

1 Materials and Instrumentation

X-ray diffraction: Single-crystal X-ray diffraction studies were performed on Bruker D8 Quest single-crystal X-ray diffractometer equipped with a graphite monochromator using Mo-K_α radiation (wavelength λ(Mo-K_α) = 0.71073 Å).

^{1}H-NMR spectroscopy: ^{1}H-NMR spectra were recorded at ambient temperature, unless otherwise stated, with a Bruker 400 MHz spectrometer. All spectra were referenced to the corresponding solvent residual signal, *i.e.* 1.940 ppm for acetonitrile, 7.260 for chloroform, 5.320 for dichloromethane and 4.870 for methanol.

FT-IR spectroscopy: FT-IR spectroscopy was performed on a Bruker Tensor 27 FT-IR spectrophotometer including a Platinum ATR unit. Signals are given as wave numbers in cm^{-1} using the following abbreviations: vs = very strong, s = strong, m = medium, w = weak and b = broad.

Elemental analysis (EA): CHN analysis was performed on a Elementar Vario MICRO cube. Pt, Mo and Mn were analyzed with inductively coupled plasma atomic emission spectroscopy (ICP-AES) on a Horiba ULTIMA 2 spectrometer.

Gas chromatography (GC): Hydrogen evolution was measured by headspace GC on a Bruker Scion GC/MS, with a thermal conductivity detector 15 (column: molecular sieve 5A, 75 m × 0.53 mm, oven temperature 70 °C, flow rate 25 ml min^{-1}, detector temperature 200 °C) using Argon as carrier gas.

Steady-State UV-Vis and emission spectroscopy: UV-Vis spectroscopy was performed on a Varian Cary 50 spectrophotometer or JASCO V-670 spectrophotometer. All systems were used with standard quartz cuvettes (d = 10.0 mm). Emission spectra were recorded on a JASCO spectrofluorometer FP-8500 at room temperature.

Time-resolved emission spectroscopy: The excitation light source for the time-resolved emission measurements is based on a Nd:YAG Laser (Continuum Surelite) yielding 5 ns pulses at 1064 nm with a repetition rate of 10 Hz. Those fundamental pulses were frequency-tripled (yielding 355 nm) which was used as input for generating the excitation pulses centered at 410 nm using an optic parametric oscillator (Continuum Surelite). After passing through the sample, the emission light was spectrally dispersed (Acton Princeton Instrument 2300), detected on a photomultiplier (Hamamatsu R928) and processed (Pascher Instruments AB). The emission kinetics were studied between 540 and 680 nm in steps of 20 nm.

Electrochemical analyses: Cyclic voltammetry (CV) experiments were performed using a CH Instruments CHI 620E electrochemical workstation equipped with a standard three electrode arrangement: glassy carbon (d = 1.6 mm) working electrode, Ag wire (in a glass frit containing electrolyte solution) quasi 2 reference electrode, Pt wire counter electrode. All potentials are quoted relative to the ferrocene/ferrocenium internal standard. CV experiments were performed in water-free, de-aerated DMF using ($n$$Bu_4N$)$PF_6$ (0.1 M) as supporting electrolyte. All solutions were purged with argon for at least 3 min to remove O_2 and kept under a slight positive Ar pressure while performing the experiment.

Computational details

All spin-polarized density functional theory (DFT) calculations were performed within the ORCA program package version 4.2.0. The geometries were initially optimized with the PBEh-3c composite method and further refined by subsequent geometry optimizations with the PBE0 hybrid functional and a def2-TZVP basis set. For molybdenum and iridium, effective core potentials were used to replace the inner 28 and 60 core electrons. In order to account for dispersion interactions, the atom-pairwise correction with the Becke-Johnson damping scheme [D3(BJ)] was utilized in all calculations. The conductor-like polarizable continuum model (CPCM) was used to implicitly include the DMF solvent environment. Electron densities were analyzed with the help of the Hirshfeld population analysis to determine atomic charges.

2 Synthesis

All chemicals were purchased from Sigma Aldrich, ABCR or ACROS and were of reagent grade. The chemicals were used without further purification unless stated otherwise. [Pt(DMSO)$_2$Cl$_2$],[1] [Pt(DMSO)$_2$I$_2$],[1] [Pt(bpy)Cl$_2$],[2] [Pt(bpy)I$_2$],[3] [Ir(ppy)$_2$(bpy)]PF$_6$,[4] and (nBu$_4$N)$_3$[MnMo$_6$O$_{18}${(OCH$_2$CNH$_2$}$_2$])$_3$,[5] were prepared according to literature procedures.

Synthesis of POM-PtCl: $(nBu_4N)_3[MnMo_6O_{18}\{(OCH_2)_3CNCH(C_{11}H_9N_2)PtCl_2\}_2]$ x 5 DMF.
$(nBu_4N)_3[MnMo_6O_{18}\{(OCH_2)_3CNCH(C_{11}H_9N_2)\}_2]$ (100 mg, 0.0446 mmol, 1 eq) and $[Pt(DMSO)_2Cl_2]$ (37.66 mg, 0.0892 mmol, 2 eq) were dissolved in water-free, de-aerated acetonitrile (10 mL). The orange solution was refluxed under argon atmosphere for 3-4 h. After cooling to room temperature, a slightly turbid solution was obtained which was filtered off and the filtrate was evaporated under vacuum to obtain a yellow precipitate. Orange crystals are formed upon slow diffusion of ethyl acetate into DMF solution. The crystals were collected by filtration and washed with diethyl ether. Yield: 70.2% based on Pt.

Elemental analysis in wt% for $C_{80}H_{140}N_9MnMo_6O_{24}Pt_2Cl_4$ (calc.): C 34.59 (34.63), H 5.19 (5.09), N 4.63 (4.54). Mw = 2774.56 g/mol.

FT-IR (in cm^{-1}):2957 (ν CH, s), 2933 (ν CH, s), 2870 (ν CH, s), 2349 (w), 1621 (ν C=N, s), 1481 (w), 1237 (w), 1089 (ν CO, m), 1021 (ν CO, s), 939 (ν Mo=O, s), 918 (ν Mo=O, s), 901 (ν Mo=O, s), 658 (ν Mo-O-Mo, vs), 563 (m), 459 (m), 410 (m).

^1H NMR: δ = 0.93 (t, 36H, nBu_4N), 1.29 (m, 24H, nBu_4N), 1.56 (m, 24H, nBu_4N), 3.15 (m, 24H, nBu_4N), 7.69 (d, 2H), 8.03 (s, 2H), 8.55 (d, 2H), 8.68 (s, 2H), 9.29 (d, 2H), 9.64 (d, 2H) ppm.

Synthesis of POM-PtI: $(nBu_4N)_3[MnMo_6O_{18}\{(OCH_2)_3CNCH(C_{11}H_9N_2)PtI_2\}_2]$.
$(nBu_4N)_3[MnMo_6O_{18}\{(OCH_2)_3CNCH(C_{11}H_9N_2)\}_2]$ (100 mg, 0.0446 mmol, 1 eq) and $[Pt(DMSO)_2I_2]$ (37.66 mg, 0.0892 mmol, 2 eq) were dissolved in dry acetonitrile (10 mL). The orange solution was refluxed under argon atmosphere for 3-4h. After cooling to room temperature, a slightly turbid solution was obtained which was filtered off and the filtrate was evaporated under vacuum to obtain a yellow precipitate. Orange crystals are formed upon slow diffusion of ethyl acetate into DMF solution. The crystals were collected by filtration and washed with diethyl ether. Yield: 76.4% based on Pt.

Elemental analysis in wt% for $C_{80}H_{140}N_9MnMo_6O_{24}Pt_2I_4$ (calc.): C 29.63 (30.60), H 4.19 (4.49), N 4.18 (4.01). Mw = 3140.37 g/mol.

FT-IR: (in cm^{-1}) 2957 (ν CH, s), 2931 (ν CH, s), 2870 (ν CH, s), 2349 (w), 1621 (ν C=N, s), 1550 (w), 1379 (m), 1089 (ν CO, m), 1021 (ν CO, s), 939 (ν Mo=O, s), 918 (ν Mo=O, s), 901 (ν Mo=O, s), 658 (ν Mo-O-Mo, vs), 563 (m), 459 (m), 410 (m).

^1H NMR: (in ppm) δ = 0.93 (t, 36H, nBu_4N), 1.32 (m, 24H, nBu_4N), 1.57 (m, 24H, nBu_4N), 3.14 (m, 24H, nBu_4N), 7.72 (d, 2H), 8.02 (d, 2H), 8.58 (s, 2H), 8.72 (d, 2H), 9.91 (d, 2H), 10.22 (s, 2H).

3 Characterization

The FT-IR spectra for both **POM-PtCl** and **POM-PtI** exhibit the characteristic bands of Anderson type POMs, *i.e.* bands between 890-950 cm^{-1} (terminal Mo=O vibrations) and bands between 660-710 cm^{-1}, which correspond to bridging Mo-O-Mo vibrations (Figure S1).[6]

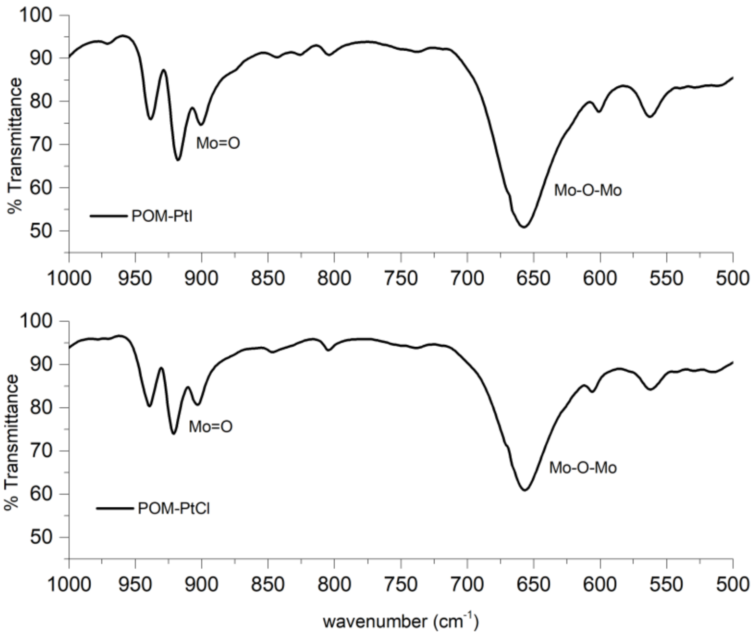

Figure S1. FT-IR spectra of **POM-PtI** (top) and **POM-PtCl** (bottom) showing the two characteristic bands for Anderson POM as indicated in the graphs.[6]

^{1}H-NMR showed the aromatic protons broadening of the bands caused by the paramagnetic Manganese center, but the chemical shift and the integrations for the aromatic protons and the aliphatic ones for both **POM-PtCl** and **POM-PtI** correspond to two bipyridine units and 3 nBu$_4$N cations.[6] Elemental analysis confirmed the bulk composition and presence of the elements indicated. Further structural characterization was provided by single-crystal X-ray diffraction (see below).

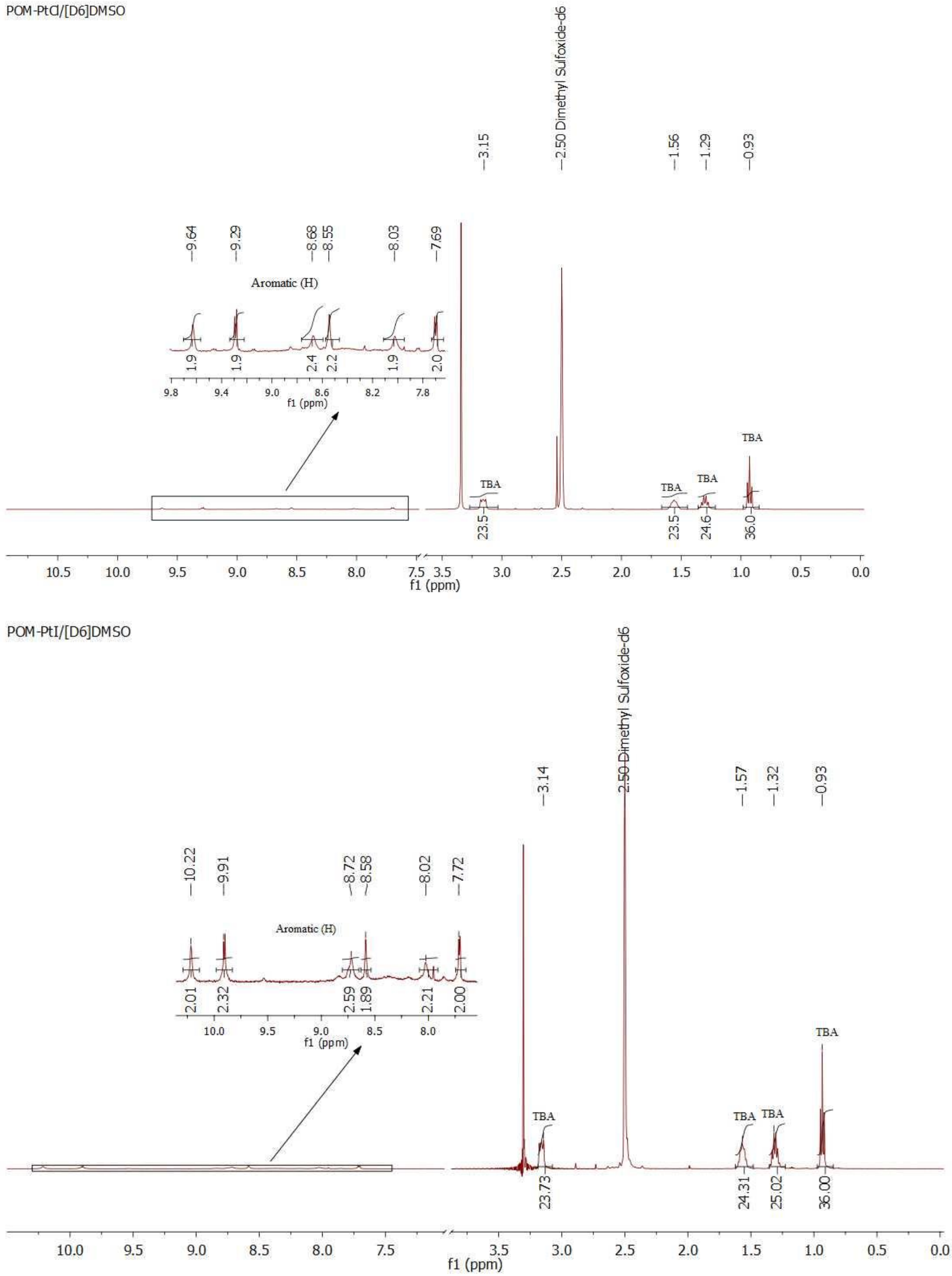

Figure S2. ^{1}H-NMR spectra of **POM-PtCl** (top) and **POM-PtI** (bottom). The spectra show the aliphatic protons of the nBu$_4$N$^+$ cations and the aromatic protons of bpy.

4. Single-crystal X-ray diffraction (scXRD) analysis of POM-PtCl and POM-PtI

Suitable single-crystals of **POM-PtCl** or **POM-PtI** were mounted onto a microloop using Fomblin oil. X-ray diffraction intensity data were measured at 150 K on a Bruker D8 QUEST diffractometer ($\lambda(Mo_{K\alpha})$ = 0.71073 Å) equipped with a graphite monochromator. Structure solution was carried out using SHELX-2013[7] package through OLEX2[8] Corrections for incident and diffracted beam absorption effects were applied using empirical methods.[9] Structures were solved by a combination of direct methods and difference Fourier syntheses and refined against F^2 by the full matrix least-squares technique. Non-hydrogen atoms were refined anisotropically. Hydrogen atoms were added to carbon atoms using a riding model. Restraints were used to model the disordered nBu_4N^+ cations. No restraints were used on the actual POM-anions. Corresponding CIF files can be obtained free of charge from the CCDC, reference numbers 2099459 (**POM-PtCl**) and 2099460 (**POM-PtI**).

Table S1 Crystallographic parameters for **POM-PtCl** and **POM-PtI**

Compound code	**POM-PtCl**	**POM-PtI**
CCDC code	2099459	2099460
Empirical formula	$C_{91}H_{155}Cl_4MnMo_6N_{11}O_{26}Pt_2$	$C_{83}H_{141}I_4MnMo_6N_{10}O_{25}Pt_2$
Formula weight / g mol^{-1}	2983.03	3207.41
Temperature / K	150	150
Crystal system	Triclinic	Monoclinic
Space group	*P*-1	*C*2/n
Unit cell dimensions / Å	a = 17.0857(6)<br>b = 18.2307(8)<br>c = 31.0330(12)	a = 38.4699(11)<br>b = 17.1588(6)<br>c = 38.4046(14)
Unit cell angles / °	α = 98.964(2)<br>β = 91.962(2)<br>γ = 100.498(2)	α =90<br>β = 95.6184(12)<br>γ = 90
Volume / Å^3	9368.9(6)	25229.0(15)
Z	3	8
Density (calcd.) / g cm^{-3}	1.586	1.689
Absorption coefficient μ / mm^{-1}	3.059	3.919
F(000)	4462	12432
2Θ range for data collection / °	3.457 to 49.424	3.978 to 52.762
Index ranges	-20 ≤ h ≤ 20, -<br>21 ≤ k ≤ 21, -36 ≤ l ≤ 36	-48 ≤ h ≤ 48, -<br>21 ≤ k ≤ 21, -47 ≤ l ≤ 48
No. reflections	166991	180729
Independent reflections	31875 [R_{int} = 0.0534,<br>R_{sigma} = 0.0346]	25823 [R_{int} = 0.0646,<br>R_{sigma} = 0.0355]
Data / restraints / parameters	18119/23/2033	25823/390/1195
Goodness-of-fit	1.061	1.075
Final R indices [I>2sigma(I)]	R_1 = 0.0612, wR_2 = 0.1622	R_1 = 0.0678, wR_2 = 0.1504
R indices (all data)	R_1 = 0.0867, wR_2 = 0.1886	R_1 = 0.0835, wR_2 = 0.11596
Largest diff. peak and hole	2.95/-3.00	1.98/-1.77

5. Electrochemistry

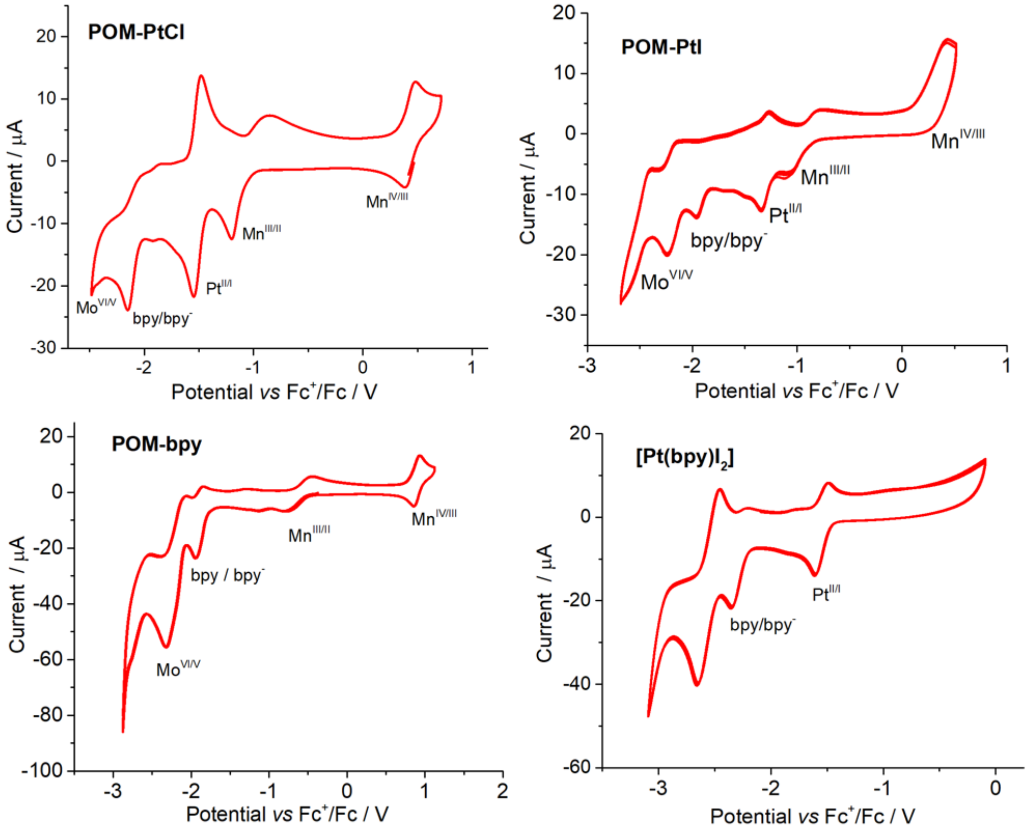

Figure S3. Cyclic voltammograms of **POM-PtCl**, **POM-PtI**, **POM-bpy and [Pt(bpy)I$_2$]** (c = 0.1 mM) in water-free, de-aerated DMF (containing 0.1 M *n*Bu$_4$NPF$_6$) at a scan rate of 0.1 Vs^{-1}. The assignments are based on literature references.[5,10–12]

Table S2: catalysis-relevant redox-potentials of the **POM-PtX** species

**Process** (tentative assignment)^a	**POM-PtCl** ***E*$_{1/2}$** / V *vs* Fc$^+$/Fc	**POM-PtI** ***E*$_{1/2}$** / V *vs* Fc$^+$/Fc
**I** (Mn$^{IV/III}$)		
**II** (Mn$^{III/II}$)	-1.02	-0.95
**III** (PtII/I)	-1.51	-1.31
**IV** (bpy/bpy$^-$)	-1.87	-1.85

^{a}based on literature data[5,10–12]

Spectro-electrochemistry. Insights into the redox activity of **POM-PtI** were obtained from spectro-electrochemical UV-Vis absorption measurements in water-free, de-aerated DMF containing 0.1 M *n*Bu$_4$NBF$_4$. Under reductive conditions (cyclic voltammetry, scan rate: 100 mVs^{-1}) two redox waves between -1.0 and -1.8 V *vs.* Fc$^{0/+}$ are observed. The UV-Vis absorption spectra monitored at the first and second reduction process, *i.e.*, at reduction potentials of -1.0 and -1.8 V *vs.* Fc$^{0/+}$. The respective spectra show slightly increased molar absorptivity between 250 and 600 nm compared to the spectrum collected without applying a potential (open-circuit-potential, 0.0 V). The difference spectrum at -1.8 V *vs.* Fc$^+$/Fc shows three major peaks at *ca*. 300, 390 and 510 nm, which are tentatively associated with a Pt$^{II/I}$ reduction, causing an increase of electron density in the bpy sphere. Due to the minor spectral changes in the investigated spectral window, the first single-electron reduction processes can be associated with the POM (*i.e.,* Mn$^{III/II}$).

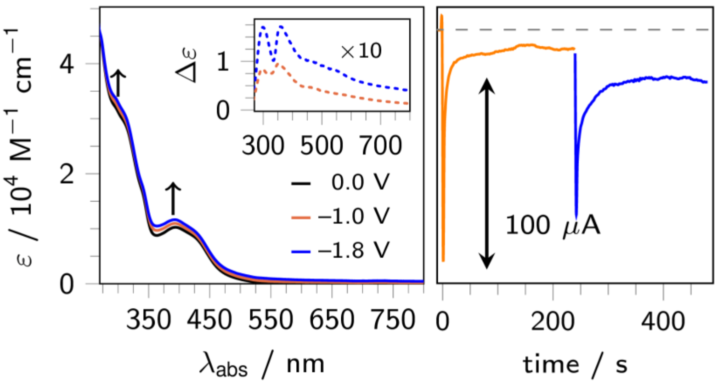

Figure S4. Left: UV-Vis absorption spectra of **POM-PtI** in water-free, de-aerated DMF and upon electrochemical reduction at -1.0 V (orange) and -1.8 V (blue) *vs.* Fc^+/Fc. Right: Chronoamperometry curves collected in the spectro-electrochemical cell. The reduction was performed under nitrogen atmosphere in water-free, de-aerated DMF containing 0.1 M $n$$Bu_4NBF_4$ (WE: GC, CE: Pt wire, pseudo-ref: Ag/Ag^+).

6. Light-driven H_2 evolution catalysis

General procedure for visible-light-driven catalytic experiments. In a typical experiment, a Schlenk tube (21 cm^3) was filled with the photosensitizer (125 µM) catalyst (12.5 µM for POM-catalysts, 25 µM for the reference catalysts), water-free, de-aerated DMF (5.7 ml), electron donor (triethylamine, 1.12 mL, 1.0 M) and a proton source (acetic acid, 0.092 ml, 0.2 M). The Schlenk tubes were irradiated with a LED light-source ((λ_{max} = 470 nm, P ~ 40 mW cm^{-2})). After irradiation, head-space gas samples (100 µL) were successively taken using a gas-tight syringe and analyzed by headspace gas chromatography. All catalytic experiments were performed in triplicate, reported values are averaged over the three runs. All catalysis experiments were performed under inert atmosphere. All solvents used for the catalysis experiments were de-gassed thoroughly by bubbling argon through the solution.

HER control experiments

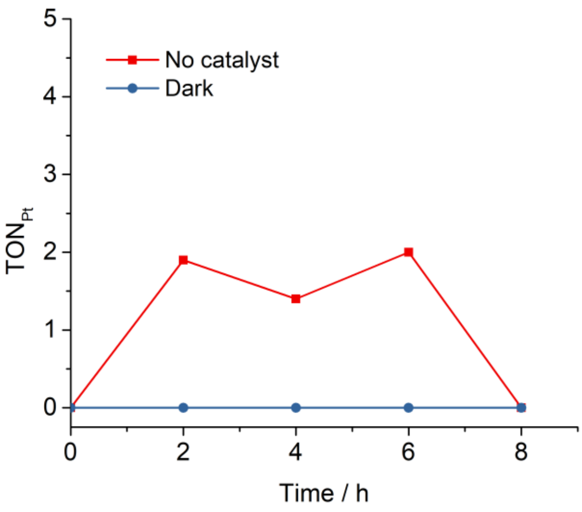

Figure S5. Hydrogen evolution control experiments in the absence of the POM-catalyst (red) and under dark, non-irradiated conditions (blue). Conditions: water-free, de-aerated DMF solution containing 125 µM **PS**, 1.0 M TEA, 0.2 M acetic acid and (dark conditions) 12.5 µM **POM-PtI**.

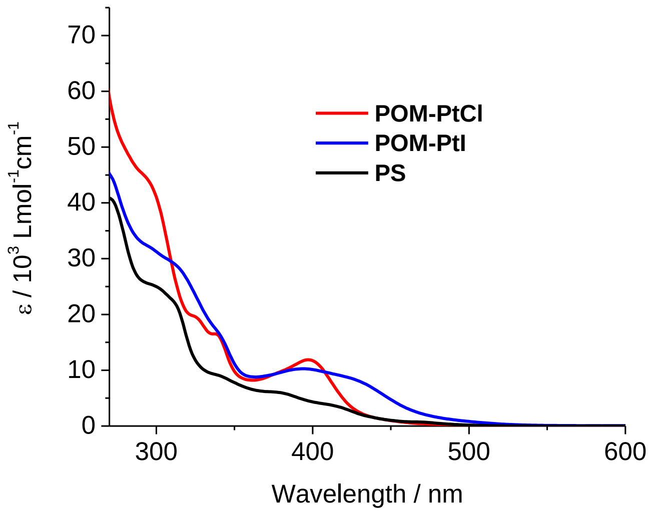

Figure S6: UV-Vis spectra for **POM-PtCl**, **POM-PtI** and **PS** $[Ir(ppy)_2(bpy)]PF_6$.

7. Mechanistic photophysical studies

Quenching mechanism studies

Steady-state fluorescence decay spectroscopy of the **PS** $[Ir(ppy)_2(bpy)](PF_6)$ was performed in the presence of various concentrations of the catalyst **POM-PtI** or the electron donor TEA in DMF solution. Before each experiment, the samples were purged with Argon for 5 min to remove dissolved oxygen. The emission intensity of $[Ir(ppy)_2(bpy)]^+$ (**PS**, 0.125 mM) between 500 and 750 nm (λ_{max} = 585 nm) upon 455 nm excitation were measured upon stepwise addition of TEA (0.1 – 1.5 M) and **POM-PtI** (6.25 – 43.75 µM).

To study the changes in the emission lifetime of **PS** upon stepwise addition of TEA or **POM-PtI**, the emission kinetics of **PS** (in aerated DMF) were monitored at 420 nm excitation between 540 and 680 nm in 20 nm steps (see Figure S6, left). The respective emission lifetimes were obtained from a global single-exponential fit of the data collected at eight emission wavelengths.

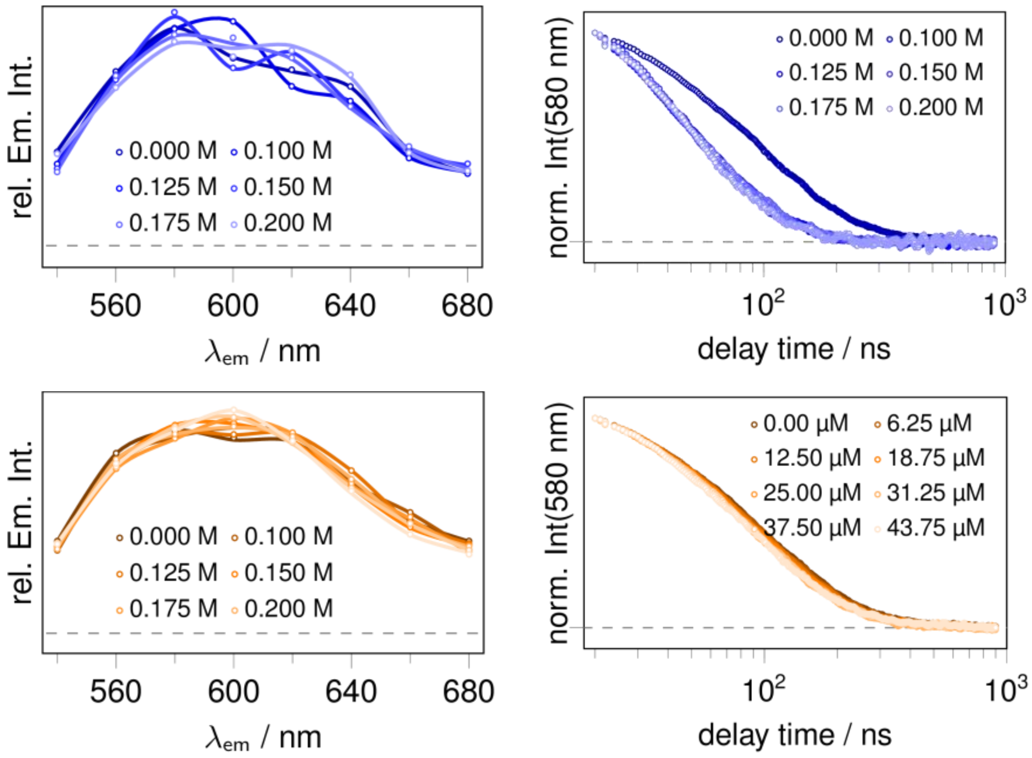

Figure S7. Emission spectra collected at a temporal delay of 35 ns (left) and kinetic traces monitored at an emission wavelength of 580 nm (right) of **PS** in DMF and upon addition of TEA (top) and **POM-PtI** (bottom).

The quenching rate constants were calculated using the Stern-Volmer relation,

$$\frac{I_0}{I} = \frac{\tau_0}{\tau} = 1 + k_q \tau_0 [Q]$$

with the emission intensity (emission lifetime) of **PS** in the absence of any quencher (I_0, τ_0 = 100 ns),[13] the intensity (emission lifetime) in presence of a quencher (I, τ), the quenching rate (k_q), and the quencher concentration ([Q]).

From the steady-state and time-resolved emission studies emission quenching rates of 8.241×10^7 $M^{-1}s^{-1}$ and 5.9×10^7 $M^{-1}s^{-1}$ for reductive quenching by TEA as well as 1.3×10^{11} $M^{-1}s^{-1}$ and 3.5×10^{10} $M^{-1}s^{-1}$ for oxidative quenching by **POM-PtI** are found. The quenching rate constants of 7.1×10^7 $M^{-1}s^{-1}$ (TEA) and 8.4×10^{10} $M^{-1}s^{-1}$ (**POM-PtI**) are calculated as averages of the quenching rate constants obtained from the steady-state and time-resolved emission studies.

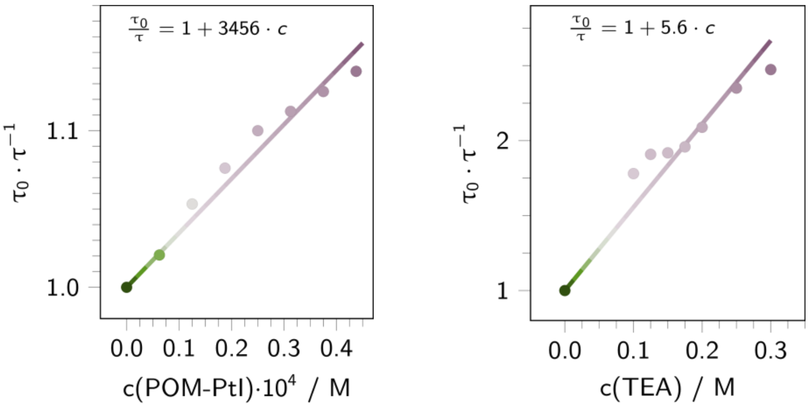

Figure S8. Stern-Volmer plots (and linear fits) for the emission lifetime quenching of $[Ir(ppy)_2(bpy)]^{+*}$ (**PS***) by **POM-PtI** (left) and TEA (right). The corresponding quenching rate constants are 3.5×10^{10} $M^{-1}s^{-1}$ (**POM-PtI**) and 5.9×10^{7} $M^{-1}s^{-1}$ (TEA), respectively. Note that a non-linear (*i.e.* quadratic) fit of the curves did not lead to meaningful values (due to negative curvature), hence a linear approximation has been chosen for quenching rate constant determination.

Determination of diffusion rate constants. According to the static quenching Collins–Kimball diffusion model a reaction occurs when the sacrificial agent reaches the immediate vicinity of the photoexcited **PS**.[14,15] The region around the **PS** is generally treated to be spherical with the radius σ obtained from the volume of the **PS**. Within the Collins–Kimball model the radius of the reaction volume R is equal to σ. Within this model the time-independent diffusion rate constants for the Ir-complex $[Ir(ppy)_2(bpy)]^{+}$ (**PS**) and triethylamine (TEA) or **POM-PtI** were calculated from

$$k_D = \frac{4 \cdot N_A \cdot k_B \cdot T \left(R_Q + R_{PS}\right)^2}{6 \cdot \eta \cdot R_Q \cdot R_{PS}},$$

using the Avogadro-constant (N_A), Boltzmann-constant (k_B), temperature (T = 298 K), the viscosity of DMF (η = 9.2×10^{-4} Pa·s) and the gyration radii of the quencher molecules R_Q (R_{TEA} =2.5×10^{-10} m, $R_{\textbf{POM-PtI}}$ = 8.7×10^{-10} m) and the Ir-photosensitizer (R_{PS} = 4.2×10^{-10} m). The gyration radii of the Ir-complex and TEA are taken from literature,[16] whereas the gyration radii of **POM-PtI** was estimated from the molecular volume (1681.98 cm^3 mol^{-1}) obtained from DFT calculations and using van-der-Waals radii from literature.[17] The resulting diffusion rate constants for **PS**/TEA and **PS/POM-PtI** in DMF are 7.7×10^{9} $M^{-1}s^{-1}$ and 8.2×10^{9} $M^{-1}s^{-1}$, respectively.

8. Computational details

Interactions between catalyst and photosensitizer

To gain insights into the interactions between catalyst and photosensitizer, the interaction energy of **PS** with the catalyst was evaluated by DFT calculations. To assess the stability of different binding sites, the interaction of the **PS** with isolated model systems was determined first. These model systems represent separate components of the **POM-PtX** catalysts (Figure S8) and serve as a reference for the interaction at the individual regions of the overall system. Various configurations of the photosensitizer with the separate bipyridine ligands (henceforth referred to as **PtCl-Ref** and **PtI-Ref**), as well as combinations with the isolated POM (**POM-Ref**) were created and geometry-optimized. The most stable reference configurations were used as initial structures for the photosensitizers at the catalyst (*i.e.* **POM-PtCl** and **POM-PtI**.). While the bipyridine systems represent neutral molecules, both **POM-Ref**, as well as **POM-PtCl** and **POM-PtI** have 3- charges. In contrast, the photosensitizer has a 1+ charge.

As shown in Figure S8, the photosensitizer attaches to the side of the reference compounds **PtCl-Ref** and **PtI-Ref**, with interaction energies of -48 kJ/mol. In the most stable configuration with the POM-Ref compound, the photosensitizer interacts at the POM's terminal oxygen atoms with an interaction energy of -76 kJ/mol. Thus, the

interaction of the **PS** with the POM is more stable than with the bipyridine systems. For **POM-PtCl** and **POM-PtI**, the more favorable interactions of the PS with the metal oxo component are even more apparent, as the photosensitizer does not remain at the bipyridine centers during geometry optimization and instead moves to the metal oxo sites. The configurations of the POM catalysts, where the photosensitizer binds to the metal oxo sites, remain stable and show an identical interaction energy as for **POM-Ref**, see Table S2.

Table S3. Interaction energies of reference and catalyst systems with the photosensitizer. The interaction energies are given in kJ/mol .

	-	X=Cl	X=I
**POM-Ref**	-76	-	-
**PtX-Ref**	-	-48	-47
**POM-PtX**	-	-77	-78

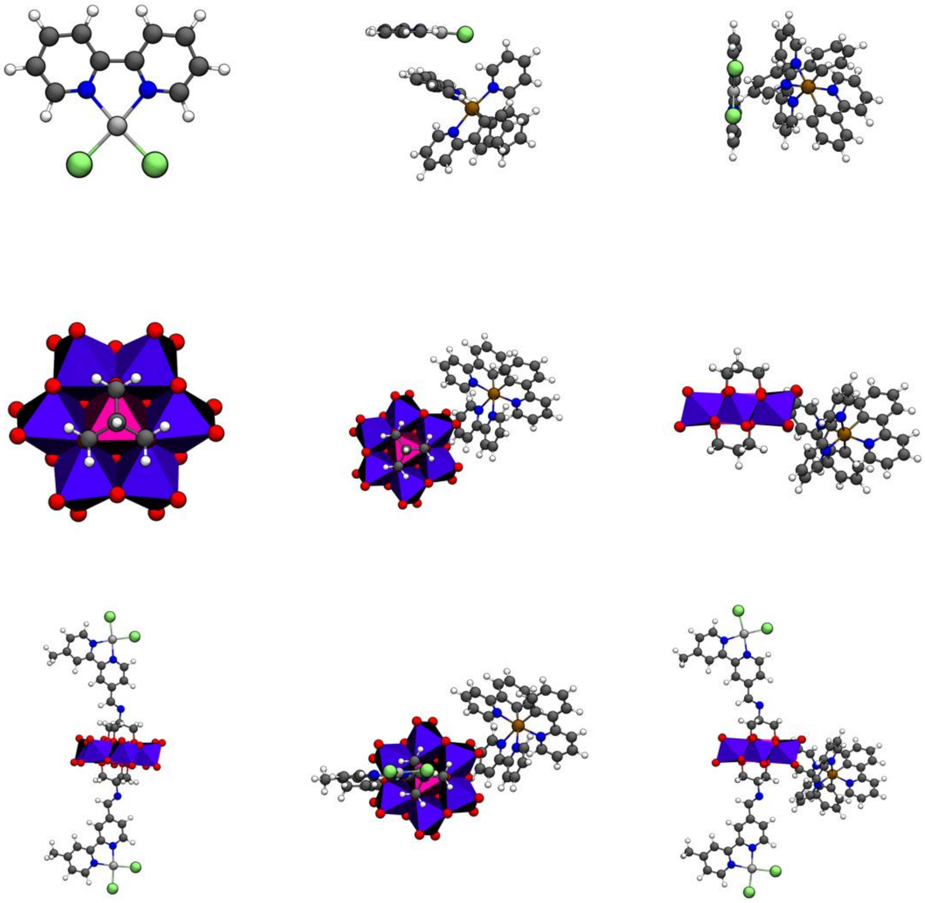

Figure S9. Representation of the investigated reference systems **PtCl-Ref**, **POM-Ref**, and combined **POM-PtCl**. Shown are the top-view and side view of the most stable aggregates of the respective compounds with the photosensitizer **PS**.

Charge analysis and distribution of electron density

To enable an estimation of how additional negative charge can be distributed over the catalysts, we performed single-point calculations with an additional electron for the reference and catalyst systems. The Hirshfeld net charges of the additionally negatively charged systems was then set in reference to the molecule's initial density. This procedure is analogous to the determination of condensed Fukui functions. While condensed Fukui

functions are normally used as descriptors for the chemical reactivity of a molecule (with respect to its electrophilic and nucleophilic regions), these functions will in this case, however, serve as a measure for the uptake of an additional electron. Here we use the condensed Fukui function $f^{+}$for the addition of an electron:

$$f^{+} = q_k(N+1) + q_k(N)$$

In this case, $q_k(N)$ describes the atomic charges at the respective atomic center k of the original system and $q_k(N+1)$ those of the system with an additional electron. In the following, the normalized values for $f^{+}$are given in percent and serve as a measure of the portion of the additional electron, which a certain region of the molecule has accepted. The corresponding values for reference and catalytic systems are shown in Table S3.

As shown in Table S3, for **PtCl-Ref** and **PtI-Ref**, > 80 % of the additional negative charge is distributed over the bpy ligands. For **POM-Ref**, most of the additional negative charge is accepted by metal-oxo framework, while the organic ligands remain unaffected. For **POM-PtCl** and **POM-PtI**, similar trends are observed as for the reference systems, and the bpy-ligands show the highest electron accepting behavior.

Table S4. Factors for the condensed Fukui function $f^{+}$for the uptake of an electron calculated via the Hirshfeld population analysis. The factors are given in percent and indicate which elements have absorbed which proportion of the additional electron.

**Element**	**PtCl-Ref**	**PtI-Ref**	**POM-Ref**	**POM-PtCl**	**POM-PtI**
H	22.74	21.04	9.70	20.94	19.46
C	49.91	47.75	1.72	44.25	42.58
N	7.43	7.28	-	10.29	10.06
Cl	14.84	-	-	13.83	-
I	-	21.27	-	-	20.00
Pt	5.57	2.66	-	4.96	2.33
O	-	-	56.04	5.45	5.29
Mn	-	-	0.28	0.08	0.08
Mo	-	-	32.82	0.21	0.19

9. Catalyst stability analyses

UV-Vis spectral changes during light-driven catalysis

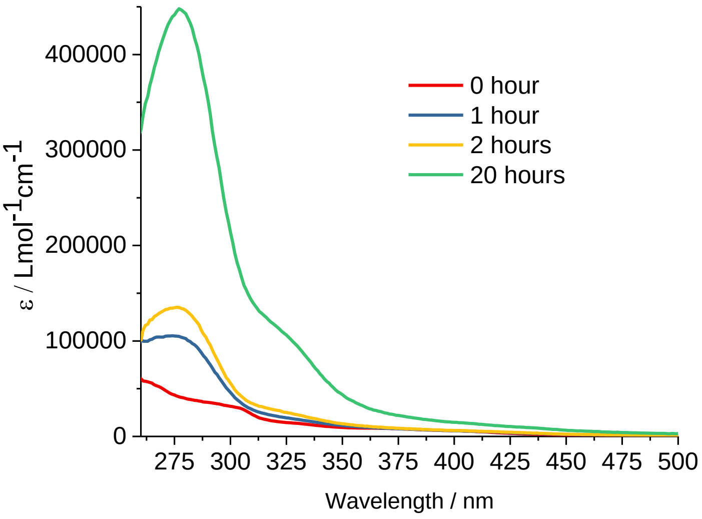

Figure S10. Development of the UV-Vis spectra for the standard **POM-PtI** catalytic mixture over 20 h irradiation. Catalysis conditions see Section 6.

UV-Vis studies on the catalyst stability. The stability of molecular catalysts under catalytic conditions is a general concern. In this context, the (photo)stability of **POM-PtI** in our system has been examined using multiple physicochemical methods. We first tested the stability of the catalyst in DMF solvent by measuring the UV-Vis spectrum of the **POM-PtI** at different time intervals. The measurements showed minor changes in the 400 – 450 nm region, which might indicate slow degradation or structural changes of the catalyst under prolonged irradiation. The exact details are still under investigation.

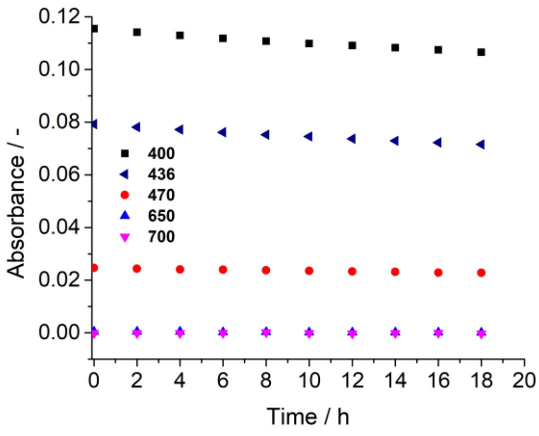

Figure S11. Time-dependent UV-Vis spectroscopic signals at characteristic **POM-PtI** absorbance maxima in DMF (400, 436, 470, 650, and 700 nm). Data collected over an 18 h period. Decrease observed: 400 nm: 6.5 %, 463nm: 7 %, 470 nm: 6 %, 650 nm: 0 %, 700 nm: 0 %)

Micro-filtration analysis. To exclude electrostatic aggregation and colloid formation between $[Ir(ppy)_2(bpy)]PF_6$/ **POM-PtI** (3-) in our reaction system, a literature-known colloid detection procedure using micro-filtration was performed:[18] A DMF solution containing $[Ir(ppy)_2(bpy)]PF_6$ and **POM-PtI** (molar ratio: 10/1) was prepared. The UV-Vis spectrum of the solution was recorded, the solution was filtered through a 0.2 µm pore size PTFE syringe filter and the UV-Vis spectrum was recorded again. UV-Vis spectroscopic analysis of the solution before and after filtration indicates no significant changes (Figure S12), suggesting that no significant number of particles have been removed from solution.

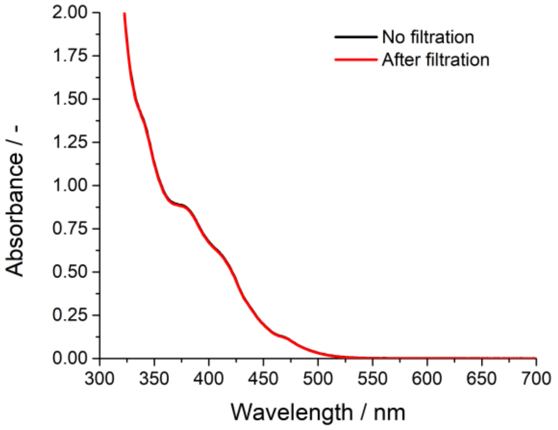

Figure S12. UV-Vis spectroscopic analysis of the reaction solution before and after micro-filtration. No changes are observed after filtration.

***In-situ* exchange of Cl- with I- ligands.** The formation of $\{PtI_2\}$ species starting from $\{PtCl_2\}$ in the presence of excess iodide is known from literature as a means to access more active HER catalysts.[19,20] Here, we used this concept to verify whether this reaction is still possible for the new **POM-PtCl** cluster. To this end, the standard light-driven HER catalysis was prepared using **POM-PtCl** as catalyst, and an excess of nBu$_4$NI (1000 fold with respect to the catalyst) was added. The standard HER catalysis was performed, and we observed a *TON* increase of ca. 20 %, indicating (at least partial) *in-situ* formation of **POM-PtI**. Further insights into this ligand exchange were obtained by UV-Vis spectroscopy: a DMF solution of **POM-PtCl** (10^{-5} M) was prepared and an excess of nBu$_4$NI (0.5 M) was added. UV-Vis spectroscopy of the solution after stirring for 48 h clearly demonstrated the conversion of **POM-PtCl** to the more active **POM-PtI** species, see below.

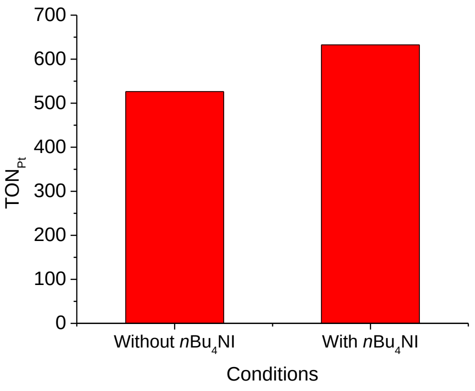

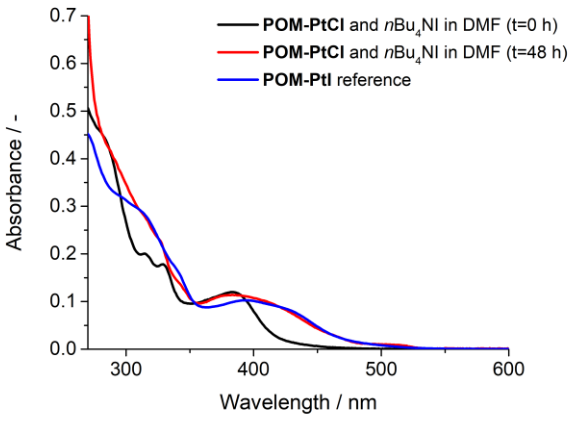

Figure S13. HER activity for **POM-PtCl** with and without excess nBu$_4$NI after 8.5 hours of irradiation (left). UV-Vis control experiment showing the substitution of Cl with I in **POM-PtCl** (right).

9. Literature references

[1] T. A. K. Al-Allaf, L. J. Rashan, A. S. Abu-Surrah, R. Fawzi, M. Steimann, *Transit. Met. Chem.* **1998**, *23*, 403–406.

[2] T. J. Egan, K. R. Koch, P. L. Swan, C. Clarkson, D. A. Van Schalkwyk, P. J. Smith, *J. Med. Chem.* **2004**, *47*, 2926–2934.

[3] S. Wimmer, P. Castan, F. L. Wimmer, N. P. Johnson, *J. Chem. Soc. Dalt. Trans.* **1989**, 403–412.

[4] A. F. Henwood, E. Zysman-Colman, *Top. Curr. Chem.* **2016**, *374*, 1–41.

[5] S. Schönweiz, S. A. Rommel, J. Kübel, M. Micheel, B. Dietzek, S. Rau, C. Streb, *Chem. Eur. J.* **2016**, *22*, 12002–12005.

[6] P. R. R. Marcoux, B. Hasenknopf, J. Vaissermann, P. Gouzerh, *Eur. J. Inorg. Chem.* **2003**, *2003*, 2406–2412.

[7] G. M. Sheldrick, *Acta Crystallogr. Sect. C Struct. Chem.* **2015**, *71*, 3–8.

[8] O. V. Dolomanov, L. J. Bourhis, R. J. Gildea, J. A. K. Howard, H. Puschmann, *J. Appl. Crystallogr.* **2009**, *42*, 339–341.

[9] R. H. Blessing, *Acta Crystallogr. A.* **1995**, *51 (Pt 1)*, 33–38.

[10] D. R. Whang, S. Y. Park, *ChemSusChem* **2015**, *8*, 3204–3207.

[11] S. Schönweiz, M. Heiland, M. Anjass, T. Jacob, S. Rau, C. Streb, *Chem. Eur. J.* **2017**, *23*, 15370–15376.

[12] D. Collison, F. E. Mabbs, E. J. L. McInnes, K. J. Taylor, A. J. Welch, L. J. Yellowlees, *J. Chem. Soc. - Dalt. Trans.* **1996**, 329–334.

[13] Y. Luo, S. Maloul, S. Schönweiz, M. Wächtler, C. Streb, B. Dietzek, *Chem. – A Eur. J.* **2020**, *26*, 8045–8052.

[14] V. S. Gladkikh, A. I. Burshtein, H. L. Tavernier, M. D. Fayer, *J. Phys. Chem. A* **2002**, *106*, 6982–6990.

[15] F. C. Collins, G. E. Kimball, *J. Colloid Sci.* **1949**, *4*, 425–437.

[16] A. Neubauer, G. Grell, A. Friedrich, S. I. Bokarev, P. Schwarzbach, F. Gärtner, A.-E. Surkus, H. Junge, M. Beller, O. Kühn, S. Lochbrunner, *J. Phys. Chem. Lett.* **2014**, *5*, 1355–1360.

[17] a. Bondi, *J. Phys. Chem.* **1964**, *68*, 441–451.

[18] B. Kirchhoff, S. Rau, C. Streb, *Eur. J. Inorg. Chem.* **2016**, *2016*, 1425–1429.

[19] S. A. De Pascali, D. Migoni, P. Papadia, A. Muscella, S. Marsigliante, A. Ciccarese, F. P. Fanizzi, *Dalton Trans.* **2006**, *2*, 5077–5087.

[20] M. González, R. Bartolomé, S. Matarraz, E. Rodríguez-Fernández, J. L. Manzano, M. Pérez-Andrés, A. Orfao, M. Fuentes, J. J. Criado, *J. Inorg. Biochem.* **2012**, *106*, 43–45.
